# Supplementary material for: Multitrait analyses identify genetic variants associated with aortic valve function and aortic stenosis risk
Source: Nat Genet. 2025 Dec 19;58(1):47–56. doi: 10.1038/s41588-025-02397-7 (PMC12807864; doi:10.1038/s41588-025-02397-7)
Supplement: Supplementary file 1 — Supplementary Results, Methods, Tables 6, 7, 10, 11, 13 and 14, and Figs. 1–9. [file 41588_2025_2397_MOESM1_ESM.pdf]

nature genetics

Supplementary information https://doi.org/10.1038/s41588-025-02397-7

Multitrait analyses identify genetic variants associated with aortic valve function and aortic stenosis risk

In the format provided by the authors and unedited

Supplementary Results 4
- Heritability and genetic correlation of velocity-encoded aortic valve phenotypes 4
- Peak Velocity and Mean Gradient 4
- Aortic Valve Area 5
- Polygenic predictions of aortic valve function linked to aortic stenosis risk in FinnGen 6
- Overlap between coronary disease and aortic valve loci 6
- Sensitivity analysis of MTAG without mean gradient 7
- Sensitivity analysis using different definitions of aortic stenosis cases 8
- Lipid scores are strong instruments to predict lipid levels 8
- PGS of lipids are associated with aortic stenosis 9
- Genetic evidence for blood sugar levels and aortic stenosis risk 9

Supplementary Methods 9
- Magnetic resonance imaging in UK Biobank 9
- Generating training and test data for segmentation 10
- Segmentation quality control 11
- Extracting velocity-encoded values 11
- Deriving phenotypes from segmentation and velocity encoding data 12
- Gene set preparation for tissue and cell-type enrichment with MAGMA 13
- The All of Us cohort description 14
- Genetic quality control and ancestry prediction in All of Us 14
- Applying polygenic scores and principal components in All of Us 15
- Phenotyping in All of Us 15
- Testing polygenic scores for association in All of Us 16
- FinnGen cohort description 17
- FinnGen ethics statement 17
- Genotyping and imputation in FinnGen 19
- Diseases and outcomes in FinnGen 19
- Aortic stenosis GWAS in FinnGen 19
- Testing polygenic scores in FinnGen 20
- Massachusetts General Brigham Biobank 20
- Phenotyping and polygenic score testing in MGB Biobank 22

UK Biobank acknowledgment 22

All of Us acknowledgment 22

MVP acknowledgment 23

FinnGen acknowledgment 23

Supplementary Tables 25
- Supplementary Table 1: Disease definitions in the UK Biobank (Excel) 25

Supplementary Table 2: Heritability of continuous aortic valve traits (Excel) 25
Supplementary Table 3: Genetic correlation of continuous aortic valve traits using REML (Excel) 25
Supplementary Table 4: GWAS top loci of continuous aortic valve traits and AS meta-analysis using METAL (Excel) 25
Supplementary Table 5: GWAS top loci of continuous aortic valve traits after excluding people with at least moderate aortic stenosis (Excel) 25
Supplementary Table 6: Baseline Characteristics UK Biobank AS cohort 25
Supplementary Table 7: Baseline Characteristics FinnGen AS cohort 26
Supplementary Table 8: Lead variants in MTAG-augmented analysis (Excel) 26
Supplementary Table 9: Overview of previous studies of aortic stenosis and replication of loci before and after MTAG (Excel) 26
Supplementary Table 10: Sensitivity analyses in UK Biobank and FinnGen 27
Supplementary Table 11: Polygenic predictions of aortic valve function and incident aortic stenosis risk in FinnGen 28
Supplementary Table 12: GWAS top loci of continuous aortic valve traits and AS meta-analysis after MTAG (Excel) 28
Supplementary Table 13: Polygenic predictions of aortic valve function and incident aortic stenosis risk after MTAG in All of Us 29
Supplementary Table 14: Polygenic predictions of aortic valve function and incident aortic stenosis risk after MTAG in Mass General Brigham Biobank 30
Supplementary Table 15: MAGMA gene set analysis (Excel) 31
Supplementary Table 16: Overview of performance of lipid based polygenic scores to predict lipid levels (Excel) 31
Supplementary Table 17: Performance of the lipid polygenic scores to predict continuous aortic valve traits (Excel) 31
Supplementary Table 18: Mendelian Randomization of risk factors and continuous aortic valve traits (Excel) 31
Supplementary Table 19: Mendelian Randomization of risk factors and aortic stenosis in METAL meta-analysis before MTAG (Excel) 31
Supplementary Table 20: Aortic stenosis definition in Mass General Biobank (Excel) 31
Supplementary Figures 32
Supplementary Figure 1: Flow diagram 32
Supplementary Figure 2: Manhattan plot of GWAS results for aortic valve traits before MTAG 34
Supplementary Figure 3: Variant effect alignment at MTAG loci 36
Supplementary Figure 4: Manhattan plot of MAGMA results for aortic valve traits 38
Supplementary Figure 5: Manhattan plot of MAGMA results for aortic stenosis 40
Supplementary Figure 6: GTEx tissue enrichment 42
Supplementary Figure 7: aortic valve cell type enrichment 44
Supplementary Figure 8: Thoracic aorta cell type enrichment 46
Supplementary Figure 9: Left ventricular cell type enrichment 48
References 50

Supplementary Results

Heritability and genetic correlation of velocity-encoded aortic valve phenotypes

We estimated SNP heritability, a measure of the variance attributable to common variant information available on the genotyping array, using BOLT-LMM[1]. These values were 36.1±1.1% for AVA, 25.3±1.1% for peak velocity, and 26.4±1.1% for mean gradient (**Supplementary Table 2**). We used BOLT-REML to calculate the genetic correlation between the aortic valve phenotypes (**Supplementary Table 3**). The genetic correlation for AVA with mean gradient was -0.56±0.019 and with peak velocity was -0.58±0.018 (**Supplementary Table 3**). The genetic correlation between mean gradient and peak velocity was 0.99±0.001.

The genetic correlation with AS based on a meta-analysis of disease-based GWAS (see **Methods**) was calculated using LDSC. The genetic correlation of this disease-based AS GWAS was 0.636±0.0033 with peak velocity, 0.635±0.033 with mean gradient, and -0.498±0.034 with AVA.

Peak Velocity and Mean Gradient

Peak velocity and AVA are the main measurements to grade severity of AS with cardiovascular imaging in clinical guidelines for valvular heart disease[2,3]. For peak velocity we found a total of 27 loci at genome-wide significance **(Supplementary Table 4 and Supplementary Figure 2)**.

The two loci, near *LPA* ($P=1.9 \times 10^{-9}$) and *GATA4* ($P=3.3 \times 10^{-22}$) have been previously reported in GWAS of aortic stenosis and bicuspid valve disease[4,5]. Bicuspid valve disease is the most common congenital heart valve disorder and affected patients are at markedly higher risk for early-onset AS and aortic root disease[6,7]. Among the loci with prior associations with related phenotypes, we identified variants near *GOSR2* ($\beta= 0.090$, $P=2.9 \times 10^{-28}$), *HMGA2* ($\beta= 0.058$, $P=2.4 \times 10^{-16}$) and *KCNRG*/*DLEU1* ($\beta=0.060$, $P=3.7 \times 10^{-17}$) that were also previously reported

for planimetric AVA[8]. Additional findings included one variant near *SMAD3* ($P=4.0 \times 10^{-8}$). *SMAD3* is associated with familial aortic aneurysm and dissection[9,10]. *SMAD3* is also known as a causal gene for the aortic Loeys-Dietz syndrome[11]. Another observation was the overlap of previously reported loci associated with coronary artery disease and those that we observed for mean gradient and peak velocity. These loci included *IMPG1/ Y RNA*, *LPA*, *SORL1*, *SURF6*, *OTUD7B*, *CTAGE1*, *LRRFIP2*, *ABCC9*, *TMEM170A* and *FERD3L/TWIST1* [12–14]. No participants in the GWAS had peak velocity greater than the threshold to define severe aortic stenosis (4 m/s), indicating that the common variant findings were driven by normal variation in the population and not an ascertainment bias.

For the highly correlated mean gradient, we found 20 loci at genome-wide significance with 17/20 loci overlapping with peak velocity and the remaining 3 loci with sub-threshold significance. These loci for mean gradient included *ABCC9*, *TMEM170A* and *ELN*. Elastin haploinsufficiency has been previously associated with aortic valve malformations[15].

Aortic Valve Area

In the GWAS for AVA we identified 43 loci at genome-wide significance including all three previously reported loci (*CRADD*, *KCNRG*/*DLEU1*, *GOSR2*) for AVA [8] **(Supplementary Table 4 and Supplementary Figure 2)**. Among these loci we observed 2 loci associated with As previously, *ACTR2* ($P=7.4 \times 10^{-10}$) and *LPA*. Rs55730499, near *LPA*—encoding lipoprotein (a) [Lp (a)]—was associated with AVA ($P=7.1 \times 10^{-15}$) with one of the biggest effect sizes ($\beta=0.082$) we observed for AVA. This variant was reported in previous GWAS of Lp (a) and is in perfect LD with rs10455872 that explains ~25% of variance of circulating Lp (a) levels[16].

Among genome-wide significant variants we found a variant near *SMAD3* ($P=7.0 \times 10^{-9}$) but also rs1840828 near *KLF2* ($P=7.4 \times 10^{-11}$) which encodes a transcription factor that has been shown to regulate hemodynamic fluid response and vascular development[17,18]. Similarly, one loci near *PKN2* ($P=8.7 \times 10^{-10}$) was observed, which encodes for protein kinase N2 that is activated by flow through the mechanoreceptor Piezo1 to regulate vascular tone[19].

Among the 31 loci we observed for AVA that were not seen for the gradient based measures, we observed further loci that were previously associated with CAD or ischemic heart disease such as *EDEM3*, *MAD2L1*, *FER*, *HMGA1*, *ATXN2* or *SLC44A2*[20–22]. Additional loci among those 31 loci were associated with aortic disease or stiffness (TGFB2, CHSY1)[23,24], cardiac fibrosis (COL8A1)[25], cardiac remodeling (CCND2, AKAP13)[26,27] or cardiac contractility (BAG3, IGFR1)[28,29].The full list of loci is provided in **Supplementary Table 4** and visualized in **Supplementary Figure 2**.

Polygenic predictions of aortic valve function linked to aortic stenosis risk in FinnGen

Polygenic predictions of AVA, peak velocity, and mean gradient were constructed using 1.1 million HapMap3 SNPs with *PRScs*[30,31]. The polygenic scores were tested in Data Freeze 12 of the external FinnGen cohort, with 6,157 cases of incident AS and 486,967 controls (**Supplementary Table 7 and 11**). The strongest associations were observed with mean gradient: a 1 standard deviation (SD) greater mean gradient score was associated with a hazard ratio (HR) of 1.23 (95% confidence interval [CI] 1.20-1.26, $P=6.5 \times 10^{-60}$) for aortic stenosis. Being in the top 5% for genetically predicted mean gradient led to an HR of 1.44 for AS (95% CI 1.35-1.54, $P=1.1 \times 10^{-27}$) compared to the bottom 95%.

Overlap between coronary disease and aortic valve loci

Out of the 61 loci identified in the analysis of aortic valve traits, 19 were among those loci identified in a recent GWAS of coronary artery disease by Aragam, et al (Supplementary Table 3 in Aragam, et al.)[12]. These loci included *ANP32E*, *OTUD7B*, *ZEB2*, *MAD2L1*, *FER*, *HMGA1*, *LPA*, *FERD3L*, *SURF6*, *PDE3A*, *ATXN2*, *TBX5*, *SMAD3*, *TMEM170A*, *GOSR2*, *CTAGE1*, *ADAMTS10*, *SLC44A2* and *KLF2*.

Similarly, out of the 134 loci identified in the MTAG-augmented analysis, 46 were also identified among the 241 loci associated with coronary artery disease in Aragam, *et al*[12]. These included

loci near *PRDM16, PCSK9, FGGY, PSRC1/CELSR2, MTMR11/OTUD7B, IL6R, ZEB2/TEX41, KALRN/UMPS, STAG1/PPP2R3A, ARHGEF26, LNX1/PDGFRA, MAD2L1, FER, SNX2, MICA/NOTCH4, LPA, FERD3L/HDAC9, TBX20, MET/CFTR, AOC1/NOS3, LPL, NSMCE2/TRIB1, TRAF1/C5, MYMK/ABO, JCAD, SUFU/CNNM2, PDE3A, CEP83/FGD6, PPP1CC/ATXN2, HNF1A, SCARB1, LIPC, SMAD3, CHRNB4/ADAMTS7, FES, TMEM170A/CFDP1, SMG6/RAP1GAP2, RPRML/GOSR3, CTAGE1, ANGPTL4, LDLR, KLF2/MYO9B, SHKBP1, APOE, ITCH/NCOA6, HORMAD2/OSM*. This list represents 34% of the MTAG-augmented loci for aortic valve measurements or aortic stenosis, and 19% of all coronary disease loci discovered to date.

Sensitivity analysis of MTAG without mean gradient

We chose to include all three measures of aortic valve function (mean gradient, peak velocity and AVA) due to the the clinical relevance of using all three measures when grading aortic valve function as recommended in the 2017 EACVI/ASE consensus document on the echocardiographic assessment of aortic stenosis severity[32]. However, due to the high genetic correction between both mean gradient and peak velocity (r=0.99±0.003), we performed a sensitivity analysis leaving out mean gradient when performing MTAG of disease-based AS meta-analysis and functional measures of aortic valve function. Compared with the main analysis, this sensitivity analysis yielded an effective sample size of N=96,365 for peak velocity (vs N=96,385), N=77,007 for AVA (vs N=77,183) and N=205,426 for AS (vs 205,483). The difference in lead SNPs was marginal; for peak velocity MSL2 was lost, for AVA CACNA1H and LCORL were gained with no losses, and for AS, CAMC2KG and EIF3A were gained with no losses. All these loci were near the significance threshold of P=5E-08 in the main analysis.

Sensitivity analysis using different definitions of aortic stenosis cases

To address the potential influence of the AS definition, we have now performed sensitivity analyses in both UK Biobank and FinnGen by using an AS definition that mandates an ICD

code for AS but also a procedural code for aortic valve surgery. In this surgery + ICD code definition (N cases = 2,053), we observe the same 4 loci in UK Biobank that we observe when only using the ICD code for AS (N cases = 3,413). In FinnGen, we observe 4 loci compared with 34 using the main analysis which is explained by the large drop in cases from 12,398 to 2,833 using the more strict definition. These four loci are replicating 3 (PALMD, ZEB3 and LPA) observed in the UK Biobank analysis (**Supplementary Table 10**).

Additionally, we investigated whether indication bias for ascertainment of AS diagnoses used for the case/control meta-analysis could majorly influence our results. To this extent, we have now performed GWAS sensitivity analyses in UK Biobank and FinnGen excluding AS cases that were preceded by a diagnosis of CAD. In UK Biobank (N cases = 3,413), we retain the same 4 loci as in the main analysis. In FinnGen, we observed 23 loci (N cases = 9,584) when excluding those with CAD before AS diagnosis compared with 34 loci (N cases = 12,398) using the main definition (**Supplementary Table 10**).

Lipid scores are strong instruments to predict lipid levels

To test the performance of the constructed lipid scores to predict lipids scores, we performed linear regression using the measured lipid traits in the cohort undergoing MRI as the outcome.

The variance of Lp (a) levels were explained better by adding the Lp (a) polygenic score in linear regression than sex, age and 5 PCs of ancestry alone (r^2=0.210 vs r^2=0.004). Similar observations were made for ApoB (r^2=0.157 vs r^2=0.016) and ApoA(r^2=0.307 vs r^2=0.174), LDL(r^2=0.132 vs r^2=0.016) and triglycerides(r^2=0.177 vs r^2=0.071) (**Supplementary Table 16**).

PGS of lipids are associated with aortic stenosis

PGS for ApoB were associated with all three valve measurements (most strongly with AVA: -0.04 SD of AVA per SD of genetically predicted ApoB, $P=8.1\times10^{-41}$; **Supplementary Table 17**). The PGS for Lp(a) was also associated with AVA, peak velocity, and mean gradient ($P=3.6\times10^{-15}$, $P=2.0\times10^{-12}$, and $P=2.7\times10^{-09}$, respectively). A PGS for triglycerides was associated with AVA, but not with peak velocity or mean gradient ($P=8.2\times10^{-21}$, P=0.31, and P=0.18, respectively), while the polygenic score for ApoA had no significant association (P=0.84, P=0.13, and P=0.11, respectively) with any trait.

Genetic evidence for blood sugar levels and aortic stenosis risk

A 486-variant genetic instrument for hemoglobin A1c—a marker of diabetes risk—was associated with a smaller AVA (β=-0.08, $P=1.8\times10^{-07}$) and greater aortic stenosis risk (OR 1.04, $P=6.0\times10^{-03}$), although this association was not statistically significant in the MR Egger or weighted median analyses (P=0.72 and 0.49, respectively, **Supplementary Table 18-19**).

Supplementary Methods

Magnetic resonance imaging in UK Biobank

The UK Biobank is a prospective, general population-based cohort study that enrolled ~500,000 individuals in the UK between the ages 40-69 years from 2006-2010[33]. Informed consent was obtained from all participants. Comprehensive phenotyping including questionnaires about family history, physical traits, life-style factors, laboratory values and imaging was obtained for each participant. Inpatient electronic health records from Hospital Episode Statistics (England), Patient Episode Database (Wales) and Scottish Morbidity Records (Scotland) as well as National Health Service death registries are linked to the cohort[34].

The imaging substudy of the UK Biobank is planned to perform 1.5 Tesla cardiac MRI in ca. 100,000 participants with ca. 65,000 studies in individual participants available as of the time of

manuscript preparation[35]. The cardiac MRI images were captured in a 20 minute study using a Siemens 1.5 Tesla MAGNETOM Aera scanner (Siemens Healthineers, Erlangen, Germany). In this study, phase contrast flow images aimed to be placed above the sinotubular junction at end-diastole were used. Phase contrast imaging uses magnitude scans as reference scans and velocity-encoded scans to create phase contrast velocity maps with a planned standard velocity encoding (VENC) of 2 m/s[36]. Over the cardiac cycle, 30 images with a slice thickness (depth) of 6 mm and a voxel size of 1.77 x 1.77 mm with retrospective gating were acquired. Consequently, the amount of time represented by each image varied among participants (due to varying heart rates).

Generating training and test data for segmentation

One cardiology fellow (S.K.) manually annotated pixels using TraceOverlay v0.1.0[37] in 1000 randomly selected images from the CINE sequences within the UK Biobank imaging series "flow_XXX_tp_AoV_bh_ePAT" within the UK Biobank (where XXX represents the velocity encoding parameter in centimeters per second). An attending cardiologist (J.P.P.) then reviewed all annotations and, as needed, adjusted them to precisely capture the aortic blood pool. J.P.P. separately manually annotated pixels of the ascending aorta in 30 additional randomly selected images for testing. For image annotation and model training, only the anatomical channel was used.

A cumulative density function (CDF) was generated from the non-background, non-lung components of the manually annotated CINE images as defined by the manually traced segmentation masks. The pixel intensities were rescaled based on the inverse of this cumulative density function, similar to the approaches described by Nyúl, et al[38], and Shinohara, et al[39]. The Kornia library[40] was then used to augment the data during training. The model was fully unfrozen and trained for 500 epochs with PyTorch using the AdamW optimizer with the

default weight decay (0.01). The training schedule was a OneCycleLR schedule, which was described by Smith and Topin for model superconvergence[41]. The loss function was a focal Dice loss with a gamma parameter of 2[42], with the focal loss component given 95% of the weight and the Dice loss component given 5% of the weight[43]. At each epoch, a Dice score was computed for the ascending aortic blood pool in the validation samples and averaged; if this average value was superior to any prior epoch, then the model weights were saved. The model weights from the epoch with the best validation Dice score (epoch 276) were saved for downstream use. The model was then applied to all available CINE images.

Segmentation quality control

After applying the model to all CINE images, the output segmentation masks underwent heuristic quality control for the ascending aortic blood pool using an approach that has been previously described[37]. Images without exactly one connected component for the ascending aortic blood pool were flagged. The instantaneous frame-to-frame change in the number of pixels attributed to the ascending aortic blood pool was computed, and any study above or below 5 standard deviations from the mean shift was flagged. Images were also flagged if both systolic and diastolic phases were not detected. Any flagged image was removed from analysis. Only participants with complete studies (those having 30 images that satisfied quality control) were retained for downstream analysis.

Extracting velocity-encoded values

Velocity at each pixel was computed within the aortic blood pool. The CINE segmentation masks were overlaid on their paired velocity-encoded images, which allowed for velocity-based measurements to be computed for labeled regions. For each aortic blood pool pixel at each of 30 time points throughout the cardiac cycle, the through-plane velocity at that pixel was extracted from the paired velocity-encoded image. For each image, the VENC value was

retrieved from the Siemens header (DICOM group 0x0029, element 0x1010). “Bits_stored" was uniformly defined as 12 in the DICOM metadata (DICOM group 0x0028, element 0x0101). Therefore, the pixel data encoded a range of intensity values from 0 through 4095 (i.e., 2^{12}-1). These were remapped to velocity values with units of centimeters per second, ranging from -VENC to +VENC, using Formula 1.

Formula 1

$$\frac{2 \times VENC \times pixel\ intensity}{2^{bits\_stored} - 1} - VENC$$

That formula yielded a through-plane velocity value for each pixel. Aggregating these values from all pixels at each frame allowed for the calculation of bulk properties such as Velocity Time Integral (VTI; necessary for computing the AVA) and forward stroke volume.

Deriving phenotypes from segmentation and velocity encoding data

Aortic diameter was computed from the CINE segmentation masks, after accounting for the physical representation of each pixel in centimeters from the DICOM metadata and computing the elliptical minor axis diameter at its largest point in systole using image moments, as previously described [37,44].

Peak velocity was determined by identifying the 99th percentile velocity for all pixels at each time point, and retaining the maximum value at any time point during systole. The 99th percentile velocity was selected as a heuristic to reduce spuriously high peak velocity values attributable to noise. The 99th percentile velocity was used to compute the gradient across the valve (using the simplified Bernoulli equation $4v^2$) for each frame, and then the mean value of that gradient at all time points during systole was taken as the mean gradient.

Forward stroke volume was computed by summing all pixel-wise forward volumes (computed by multiplying velocity, width, height, and duration of each frame) for the aortic blood pool during systole.

Aortic valve area was computed by dividing forward stroke volume by VTI [45]. The 99th percentile pixel velocity was treated as the boundary of the VTI envelope throughout the cardiac cycle in order to calculate VTI for this formula.

Gene set preparation for tissue and cell-type enrichment with MAGMA

Tissue data were taken from GTEx v8[46,47]. scRNA-seq data for left ventricular cardiomyocytes were taken from Chaffin, et al[48] after reprocessing by PlaqView[49]. Ascending aortic scRNA-seq data were taken from Pirruccello, et al[37] via the Broad Institute single cell portal with accession #SCP1265.

GTEx v8 RNA sequencing gene reads were combined with the GTEx sample attributes metadata and then processed in EdgeR[50]. The edgeR::DGEList function was called to ingest the data. Then the edgeR::calcNormFactors function was called for normalization. Genes with an expression count of less than 100 were then removed. The data were loaded into voom[51]. Iterating over each tissue as a target (with weight 1), all other tissues were considered non-target and weighted by -1 times the number of samples contributing to that tissue divided by the total number of samples. Tissues with the same prefix (e.g., “Artery”) were excluded from the non-target tissue set for each target that had a prefix. The design matrix compared expression between the non-target tissues (assigned to negative weights) and the target tissue (assigned to weight +1), with model fitting performed by voom::eBayes. A gene set was created for each target tissue by filtering to keep genes with log fold change between target and non-target tissues of greater than two and false discovery rate (FDR)-adjusted P-value < 0.01.

The other gene sets all contained information about the log fold change of each gene for each cell type (or condition) and were filtered to ensure a positive log-fold change, gene presence in greater than or equal to 5 percent of cells, and FDR-adjusted P-value < 0.05. Author labels for cell types or conditions were used where provided; otherwise, numerical Seurat clusters provided by PlaqView were used. The gene list for each cell type (or condition) for each study was treated as its own gene set for MAGMA gene-set analysis.

The *All of Us* cohort description

The *All of Us* research program is a multi-site, prospective cohort study in the United States [52]. The enrollment process included a physical examination and biospecimen collection, with follow-up based on electronic health record (EHR) records and surveys. All participants provided written, informed consent. Analysis of *All of Us* was considered exempt by the UCSF IRB (#22-37715).

Genetic quality control and ancestry prediction in *All of Us*

At the time of analysis, whole genome sequencing (WGS) had been completed in 98,622 participants. Sequencing and sample quality control in All of Us has been detailed previously [53]. In brief, sequencing was performed with Illumina NovaSeq 6000. Alignment to GRCh38 and variant calling was performed with DRAGEN v3.4.12.

Analyses in this manuscript were conducted using data from release C2022Q2R6. Sample-level quality control was performed centrally by All of Us, with steps that are summarized here. Samples were excluded if (a) fingerprint concordance log likelihood ratio was <= -3 (to detect sample swaps and contamination); (b) sex reported at birth was either “Male” or “Female” and was discordant with the WGS-based chromosomal sex call (to detect sample swaps); (c) contamination rate was >= 3% (to detect contamination); or (d) mean coverage was < 30x, or <90% of bases had 20x coverage, there were <8e10 aligned Q30 bases, or <95% of bases in

59 hereditary disease risk genes had 20x coverage. Fingerprint concordance was checked at 114 sites using Picard v2.23.9.

After sample-level quality control, a joint callset was created and quality controlled centrally by *All of Us*[3]. Variants were filtered if there was no high quality genotype at the site, if ExcessHet for the site was <54.69, or if the QUAL score was <60 for SNPs or <69 for Indels. 657,238,701 variant sites were retained.

Ancestry prediction was performed centrally by *All of Us*; briefly, Human Genome Diversity Project and 1000 Genomes samples were used to train a random forest to identify ancestry labels based on PCA from high-quality variant sites, and these loadings were then applied in *All of Us*.

Applying polygenic scores and principal components in *All of Us*

PRScs-based polygenic score weights were lifted over from GRCh37 to GRCh38 using the hg19ToHg38 UCSC chain file[54]. Polygenic scores were then applied to all participants with WGS as an allelic sum. To produce principal components of ancestry (PCA), WGS calls were filtered to retain minor allele frequency (MAF) >= 0.01 and maximum variant call missingness < 0.01; variants were pruned within a 1-megabase window with maximum r^2 0.01; and then PCA was computed with Plink2[55] using the --pca approx allele-wts option. The loadings were applied to all participants with WGS data. To avoid inflation in the prediction effect sizes due to ancestry differences that may be reflected in the score weights, each score was residualized for the first 20 principal components of ancestry.

Phenotyping in *All of Us*

AS was defined to be present starting on the date of the first of the following diagnostic or procedural codes. SNOMED: 60573004, 194987006, 194984004, 194733006, 194735004, 427515002, 19833008, 836482000, 26212005, 725351001; ICD10PCS: 02RF38Z, 02RF08Z,

02RF0JZ, 027F3ZZ, 02QF0ZZ, X2RF032, X2RF332, 02RF3JZ, 02RF3KZ, 02RF48Z, 02RF4JZ; ICD9Proc: 35.21, 35.22, 35.06; or CPT4: 33405, 33361, 33412, 33366, 92986, 0318T, 33362, 33363, 33365, 33367, 33369, 33400, 33406, 33410, 33411.

AVA measurements based on the continuity equation using the velocity time integral (VTI) and cross-sectional area from echocardiography were identified using the *All of Us* data browser. For participants with multiple measurements, the first was retained. Participants were excluded from downstream analysis if identified as having AS prior to their first measurement.

Testing polygenic scores for association in All of Us

The PCA-residualized polygenic scores were tested for association with the presence or absence of the disease label of AS, and separately for association with the continuous phenotype of AVA. Statistical analyses were conducted using R 4.2.2.

The presence or absence of AS was tested as a binary outcome using the following independent variables: the polygenic score, age at the time of *All of Us* data pull (for C2022Q2R6 this date was 2022-01-01), whether sex at birth was assigned “Male,” the interaction of age and sex, and height at enrollment. (Height, but not weight or other anthropometrics, was included because we expect it to remain approximately constant throughout adulthood.) All continuous variables were scaled to mean 0 and standard deviation 1. In a linear mixed model-based analysis using the *lme4* package (and the *lmerTest* package to compute P values), ancestry was used as a random effect. Principal components were not included in the linear mixed model due to singular values. Two fixed-effect logistic models were also computed. In the first, all ancestries were retained and PC1-5 were used to account for ancestry. In the second, only individuals in the largest ancestry group (“EUR”) were retained, and PC1-5 were still included.

AVA as a continuous trait was tested using the same independent variables as described above. Because the sample size with echocardiography data was small, we excluded any group with predicted ancestry sample size of fewer than 100 participants, leaving only AFR, AMR, and EUR groups for this analysis. The modeling strategy was similar to the AS analysis. The linear mixed model approach with ancestry as random effect but no PCs was tested; a linear model with all ancestries and PCs was tested; and a linear model with only EUR participants and PCs was tested.

FinnGen cohort description

FinnGen is a nationwide public-private research project combining genomic and digital healthcare data for Finnish individuals, aiming to provide novel medically and therapeutically relevant insights into human diseases. The FinnGen data used in this study comprise 500,348 individuals from FinnGen Data Freeze 12 (https://www.finngen.fi/en).

FinnGen ethics statement

Patients and control subjects in FinnGen provided informed consent for biobank research, based on the Finnish Biobank Act. Alternatively, separate research cohorts, collected prior the Finnish Biobank Act came into effect (in September 2013) and start of FinnGen (August 2017), were collected based on study-specific consents and later transferred to the Finnish biobanks after approval by Fimea (Finnish Medicines Agency), the National Supervisory Authority for Welfare and Health. Recruitment protocols followed the biobank protocols approved by Fimea. The Coordinating Ethics Committee of the Hospital District of Helsinki and Uusimaa (HUS) statement number for the FinnGen study is Nr HUS/990/2017.

The FinnGen study is approved by Finnish Institute for Health and Welfare (permit numbers: THL/2031/6.02.00/2017, THL/1101/5.05.00/2017, THL/341/6.02.00/2018, THL/2222/6.02.00/2018, THL/283/6.02.00/2019, THL/1721/5.05.00/2019 and

THL/1524/5.05.00/2020), Digital and population data service agency (permit numbers: VRK43431/2017-3, VRK/6909/2018-3, VRK/4415/2019-3), the Social Insurance Institution (permit numbers: KELA 58/522/2017, KELA 131/522/2018, KELA 70/522/2019, KELA 98/522/2019, KELA 134/522/2019, KELA 138/522/2019, KELA 2/522/2020, KELA 16/522/2020), Findata permit numbers THL/2364/14.02/2020, THL/4055/14.06.00/2020, THL/3433/14.06.00/2020, THL/4432/14.06/2020, THL/5189/14.06/2020, THL/5894/14.06.00/2020, THL/6619/14.06.00/2020, THL/209/14.06.00/2021, THL/688/14.06.00/2021, THL/1284/14.06.00/2021, THL/1965/14.06.00/2021, THL/5546/14.02.00/2020, THL/2658/14.06.00/2021, THL/4235/14.06.00/2021, Statistics Finland (permit numbers: TK-53-1041-17 and TK/143/07.03.00/2020 (earlier TK-53-90-20) TK/1735/07.03.00/2021, TK/3112/07.03.00/2021) and Finnish Registry for Kidney Diseases permission/extract from the meeting minutes on 4th July 2019.

The Biobank Access Decisions for FinnGen samples and data utilized in FinnGen Data Freeze 12 include: THL Biobank BB2017_55, BB2017_111, BB2018_19, BB_2018_34, BB_2018_67, BB2018_71, BB2019_7, BB2019_8, BB2019_26, BB2020_1, BB2021_65, Finnish Red Cross Blood Service Biobank 7.12.2017, Helsinki Biobank HUS/359/2017, HUS/248/2020, HUS/150/2022 § 12, §13, §14, §15, §16, §17, §18, and §23, Auria Biobank AB17-5154 and amendment #1 (August 17 2020) and amendments BB_2021-0140, BB_2021-0156 (August 26 2021, Feb 2 2022), BB_2021-0169, BB_2021-0179, BB_2021-0161, AB20-5926 and amendment #1 (April 23 2020) and its modification (Sep 22 2021), Biobank Borealis of Northern Finland_2017_1013, 2021_5010, 2021_5018, 2021_5015, 2021_5023, 2021_5017, 2022_6001, Biobank of Eastern Finland 1186/2018 and amendment 22 § /2020, 53§/2021, 13§/2022, 14§/2022, 15§/2022, Finnish Clinical Biobank Tampere MH0004 and amendments (21.02.2020 & 06.10.2020), §8/2021, §9/2022, §10/2022, §12/2022, §20/2022, §21/2022, §22/2022, §23/2022, Central Finland Biobank 1-2017, and Terveystalo Biobank STB 2018001 and amendment 25th Aug 2020, Finnish Hematological Registry and Clinical Biobank decision 18th June 2021, Arctic biobank P0844: ARC_2021_1001.

Genotyping and imputation in FinnGen

Newly collected FinnGen samples were genotyped using a FinnGen ThermoFisher Axiom custom array (Thermo Fisher Scientific, San Diego, CA, USA), and legacy cohorts were genotyped using Illumina and Affymetrix arrays (Illumina Inc., San Diego, and Thermo Fisher Scientific, Santa Clara, CA, USA) as detailed previously[56]. Samples who were twins or duplicates of included samples were removed, and samples who were not of Finnish ancestry based on genomic principal component analysis were further excluded. Genotype imputation was performed using a population-specific SISu v4 imputation reference panel comprised of 8,557 whole genomes and using the Beagle v4.1 software according to the protocol available at: https://dx.doi.org/10.17504/protocols.io.xbgfijw.

Diseases and outcomes in FinnGen

Data for all individuals were linked by unique national personal identification numbers to the national hospital discharge registry and the cause of death registry (available from 1969). Aortic stenosis cases were identified based on at least one registered instance according to the ICD-10 (codes I35.0 and I35.2) or ICD-9 (codes 4241B and 4241C) classifications. Additionally, control participants were excluded if they had any of the following NOMESCO Classification of Surgical Procedures codes corresponding to aortic valve operations: FMA*, FMB*, FMD0[0-2]*, FMD15, FMD96, FME05, and FME[123467]0.

We additionally evaluated a secondary aortic stenosis case definition, limiting cases to those participants who had at least one procedural code corresponding to “Replacement of aortic valve” (NOMESCO code category FMD*) or “Repair of aortic valve for stenosis” (NOMESCO code category FMA*) in addition to an ICD code for aortic stenosis.

Aortic stenosis GWAS in FinnGen

We conducted case-control GWAS of aortic stenosis in FinnGen using REGENIE v2.2.4, with sex, age at death or end of follow-up, principal components 1–10, genotyping array, and genotyping batch as fixed-effect covariates. An approximate Firth's correction was used for variants reaching nominal P<0.01 in an initial test; standard errors were computed from Firth's beta estimates and Firth's P-values. The primary GWAS of ICD-based aortic stenosis included 12,398 cases and 487,930 controls. The secondary GWAS of ICD-based aortic stenosis with procedural intervention included 2,833 cases and 487,930 controls.

Testing polygenic scores in FinnGen

Polygenic scores were applied to all participants with imputed genotype data as an allelic sum using Plink2[55], and subsequently tested for association with incident aortic stenosis after DNA collection after excluding participants with prevalent aortic stenosis. Survival analysis was performed using Cox proportional hazards models with the follow-up time scale as implemented in the *survival* package (version 3.2.7) in R (version 4.3.2). Follow-up time was defined as the time from DNA collection to diagnosis of aortic stenosis for cases, and as the time from DNA collection to end of follow-up, death, or emigration abroad for controls. Covariates included self-reported sex, age at DNA collection, (age at DNA collection)^2, the genotyping array, and the first five principal components of genetic ancestry. Proportional hazards assumptions were tested by evaluation of Schoenfeld residuals.

Massachusetts General Brigham Biobank

The Massachusetts General Brigham Biobank (MGB) is an ongoing observational biobank enrolling participants from a multicenter health system in Massachusetts, USA[57]. Participants are enrolled with broad-based consent collected by local research coordinators, either as part of a collaborative research study or electronically through a patient portal[58]. Demographic data, blood samples and surveys are collected at baseline and linked to electronic health record data.

All adult patients provided informed consent to participate. A small number of children were enrolled with IRB-approved assent forms; upon reaching 18 years of age all enrolled children had to provide consent or were removed from the study. The Human Research Committee of MGB approved the Biobank protocol (2009P002312). Exome sequencing and array genotyping have been completed for over 53,000 MGB participants. Samples were exome sequenced on Illumina NovaSeq machines with a custom exome panel (TWIST Human Core Exome), with a target of at least 20X coverage at >85% of target sites. The Genome Analysis Toolkit (GATK v4.1) was employed for alignment, processing, and joint-calling of variants, following GATK recommended practices. A stringent quality control (QC) process was applied post-sequencing, closely following a previously published pipeline[59].

All samples were also genotyped using the GlobalScreeningArray version 1. The genotype array data underwent stringent QC. Variant QC consisted of removal of variants with allele count <2, missingness >2%, Hardy Weinberg equilibrium test *P*-value <1e-6 (in each continental super-population), and those with discordant frequencies as compared to gnomAD (X^2 statistic >300; applied in each continental super-population). Sample QC consisted of removal of outliers for heterozygosity or missingness, removal of samples with a mismatch between inferred and self-reported sex, and removal of samples with a mismatch between exome sequencing and array calls. Principal component analysis and relatedness inference were performed using PC-Relate[60] and PC-Air[61], while ancestry labels (for continental super-populations) were learned from a k-nearest-neighbor model trained on 1000Genomes project data[62]. Following these stringent QC procedures, data were subsequently genome-wide imputed to the TOPMed imputation panel (r2) on the Michigan Imputation Server (submitted by batch)[63]. These imputed data were used in downstream analyses of PRS; information sufficient for downstream analysis on aortic valve code status was available for 44,326 unrelated participants.

Phenotyping and polygenic score testing in MGB Biobank

The participants were defined as having aortic stenosis by using ICD codes and Current Process Terminology (CPT) codes when at least one of the specified codes was registered in the electronic health records of MGB (**Supplementary Table 20**). Polygenic scores were applied to all participants with imputed genotype data as an allelic sum using Plink2[55]. We performed Cox proportional hazards models for incident AS, adjusting for sex, age at DNA collection, $(\text{age at DNA collection})^2$, the first five principal components, to calculate the odds ratio per one standard deviation of polygenic risk. Additionally, the principal components 1 to 10 were regressed out of the PRS values. The time of follow up was defined as the time from DNA collection. We also performed a stratified analysis comparing the top 5% of participants based on polygenic risk against the remaining participant pool, using the same set of adjustments in Cox proportional hazard models.

UK Biobank acknowledgment

UK Biobank was accessed under application #41664. UK Biobank is generously supported by its founding funders the Wellcome Trust and UK Medical Research Council, as well as the British Heart Foundation, Cancer Research UK, Department of Health, Northwest Regional Development Agency and Scottish Government.

All of Us acknowledgment

The *All of Us* Research Program is supported by the National Institutes of Health, Office of the Director: Regional Medical Centers: 1 OT2 OD026549; 1 OT2 OD026554; 1 OT2 OD026557; 1 OT2 OD026556; 1 OT2 OD026550; 1 OT2 OD 026552; 1 OT2 OD026553; 1 OT2 OD026548; 1 OT2 OD026551; 1 OT2 OD026555; IAA #: AOD 16037; Federally Qualified Health Centers: HHSN 263201600085U; Data and Research Center: 5 U2C OD023196; Biobank: 1 U24

OD023121; The Participant Center: U24 OD023176; Participant Technology Systems Center: 1 U24 OD023163; Communications and Engagement: 3 OT2 OD023205; 3 OT2 OD023206; and Community Partners: 1 OT2 OD025277; 3 OT2 OD025315; 1 OT2 OD025337; 1 OT2 OD025276. The *All of Us* Research Program would not be possible without the partnership of its participants.

MVP acknowledgment

The authors thank Million Veteran Program (MVP) staff, researchers, and volunteers, who have contributed to MVP, and especially participants who previously served their country in the military and now generously agreed to enroll in the study[64]. This research is based on data from the Million Veteran Program, Office of Research and Development, Veterans Health Administration, and was supported by the Veterans Administration MVP award #000.

FinnGen acknowledgment

We want to acknowledge the participants and investigators of FinnGen study. The FinnGen project is funded by two grants from Business Finland (HUS 4685/31/2016 and UH 4386/31/2016) and the following industry partners: AbbVie Inc., AstraZeneca UK Ltd, Biogen MA Inc., Bristol Myers Squibb (and Celgene Corporation & Celgene International II Sàrl), Genentech Inc., Merck Sharp & Dohme LCC, Pfizer Inc., GlaxoSmithKline Intellectual Property Development Ltd., Sanofi US Services Inc., Maze Therapeutics Inc., Janssen Biotech Inc, Novartis Pharma AG, and Boehringer Ingelheim International GmbH. Following biobanks are acknowledged for delivering biobank samples to FinnGen: Auria Biobank (www.auria.fi/biopankki), THL Biobank (www.thl.fi/biobank), Helsinki Biobank (www.helsinginbiopankki.fi), Biobank Borealis of Northern Finland (https://www.ppshp.fi/Tutkimus-ja-opetus/Biopankki/Pages/Biobank-Borealis-briefly-in-English.aspx), Finnish Clinical Biobank Tampere (www.tays.fi/en-

US/Research_and_development/Finnish_Clinical_Biobank_Tampere), Biobank of Eastern Finland (www.ita-suomenbiopankki.fi/en), Central Finland Biobank (www.ksshp.fi/fi-FI/Potilaalle/Biopankki), Finnish Red Cross Blood Service Biobank (www.veripalvelu.fi/verenluovutus/biopankkitoiminta), Terveystalo Biobank (www.terveystalo.com/fi/Yritystietoa/Terveystalo-Biopankki/Biopankki/) and Arctic Biobank (https://www.oulu.fi/en/university/faculties-and-units/faculty-medicine/northern-finland-birth-cohorts-and-arctic-biobank). All Finnish Biobanks are members of BBMRI.fi infrastructure (www.bbmri.fi). Finnish Biobank Cooperative -FINBB (https://finbb.fi/) is the coordinator of BBMRI-ERIC operations in Finland. The Finnish biobank data can be accessed through the Fingenious® services (https://site.fingenious.fi/en/) managed by FINBB.

Supplementary Tables

Supplementary Table 1: Disease definitions in the UK Biobank (Excel)

Supplementary Table 2: Heritability of continuous aortic valve traits (Excel)

Supplementary Table 3: Genetic correlation of continuous aortic valve traits using REML (Excel)

Supplementary Table 4: GWAS top loci of continuous aortic valve traits and AS meta-analysis using METAL (Excel)

Supplementary Table 5: GWAS top loci of continuous aortic valve traits after excluding people with at least moderate aortic stenosis (Excel)

Supplementary Table 6: Baseline Characteristics UK Biobank AS cohort

	AS Case	Control	All
N	5,038	41,2301	417,339
Age at enrollment	63.3 (5.6)	57.1 (8.1)	57.2 (8.1)
Age at censoring	75.1 (5.8)	69.4 (8.1)	69.4 (8.1)
Female	1,833 (36.4%)	225,402 (54.7%)	227,235 (54.4)
Body mass index (kg/m^2)	29.7 (5.5)	27.5 (4.8)	27.5 (4.8)
Hypertension	4,117 (81.7%)	162,226 (39.3%)	166,343 (39.9%)
Type 2 Diabetes	1,418 (28.1%)	34,859 (8.5%)	36,277 (8.7%)
Atrial Fibrillation	2,182 (43.3%)	30,251 (7.3%)	32,433 (7.8%)
Coronary Artery Disease	3,149 (62.5%)	43,961 (10.7%)	47,110 (11.3%)
AV intervention	2,086 (41.4%)	509 (0.1%)	2,595 (0.6%)

AS: aortic stenosis, AV: aortic valve

Supplementary Table 7: Baseline Characteristics FinnGen AS cohort

	AS Case	Control	All
N	12,398	487,930	500,328
Age at DNA sampling	68.77 (12.22)	52.71 (17.83)	53.11 (17.89)
Age at end of follow-up	78.39 (10.20)	60.37 (17.96)	60.81 (18.03)
Female	4,684 (37.8)	277,370 (56.8)	282,054 (56.4)
Body mass index (kg/m^2)	28.11 (4.99)	27.32 (5.54)	27.35 (5.53)
Hypertension	8,928 (72.0)	145,695 (29.9)	154,623 (30.9)
Type 2 Diabetes	4,716 (38.0)	86,278 (17.7)	90,994 (18.2)
Atrial Fibrillation	5,673 (45.8)	57,854 (11.9)	63,527 (12.7)
Coronary Artery Disease	4,960 (40.0)	51,685 (10.6)	56,645 (11.3)
Aortic Stenosis	12,398 (100.0)	0 (0.0)	12,398 (2.5)
AV Intervention	2,833 (22.9)	453 (0.1)	3,286 (0.7)

AS: aortic stenosis, AV: aortic valve

Supplementary Table 8: Lead variants in MTAG-augmented analysis (Excel)

Supplementary Table 9: Overview of previous studies of aortic stenosis and replication of loci before and after MTAG (Excel)

Supplementary Table 10: Sensitivity analyses in UK Biobank and FinnGen

Cohort	UK Biobank, all	UK Biobank, No CAD	UK Biobank, Surgical AS	FinnGen, all	FinnGen, No CAD	FinnGen, Surgical AS
N Cases	5,038	3,413	2,053	12,398	9,584	2,833
N Controls	412,301	398,814	414,743	487,930	487,930	487,930
N Lead SNPs	4	4	4	34	23	4
Nearest Genes	PALMD, ZEB2, LPA, IL6	PALMD, ZEB2, LPA, IL6/STEAP1B	PALMD, ZEB2, LPA, IL6	LMO4, PALMD, ADAMTSL4, PRRX1, NAV1, DSTYK, RNF144A, ZEB2, TMEM44, AC058822.1, FER, SNCAIP, TSBP1, NMBR, LPA, IL6, FERD3L, FADS1/FADS2, MYEOV, FGF23, PDE3A, PLXNC1, SMAD9, STARD9, SPG11, CHRNB4, GLIS2, CFDP1, MEOX1, CA10, BAHCC1, TSPAN16, HORMAD2, RPS6KA3	ALPL, PALMD, PRRX1, NAV1, RNF144A, ZEB2, TMEM44, AC058822.1/PDGFRA, FER, TSBP1, NMBR, LPA, DGKB, IL6, FADS1/FADS2, MYEOV, PDE32, STARD9, TRIM69, BCAR1, MEOX1, BAHCC1, TSPAN16	PALMD, RNF144A, ZEB2, LPA

CAD: coronary artery disease, AS: aortic stenosis, SNP: single nucleotide polymorphism

Supplementary Table 11: Polygenic predictions of aortic valve function and incident aortic stenosis risk in FinnGen

Standard deviation scaled PRS as predictor

PRS	Hazard ratio for incident aortic stenosis	P Value
AVA	0.81 (0.79–0.83)	4.3E-59
Peak velocity	1.24 (1.21–1.27)	1.2E-62
Mean gradient	1.23 (1.20–1.26)	6.5E-60

PRS status as predictor (Top 5% vs others)

PRS	Hazard ratio for incident aortic stenosis	P Value
AVA	0.70 (0.63–0.78)	1.3E-11
Peak velocity	1.43 (1.34–1.53)	1.3E-26
Mean gradient	1.44 (1.35–1.54)	1.1E-27

Polygenic predictions of aortic valve area (AVA), peak velocity, and mean gradient were constructed using 1.1 million HapMap3 SNPs with *PRSCs* and tested in FinnGen using Cox proportional hazard models. After exclusion of prevalent aortic stenosis (AS) cases at the time of DNA sampling, 4,585 cases of incident AS and 399,194 controls contributed to survival analyses with the follow-up time scale. Covariates included self-reported sex, age at DNA collection, age^2 at DNA collection, the genotyping array, and the first five principal components of genetic ancestry. Results are shown separately for standard deviation scaled and percentile-stratified PRS as predictors.

Supplementary Table 12: GWAS top loci of continuous aortic valve traits and AS meta-analysis after MTAG (Excel)

Supplementary Table 13: Polygenic predictions of aortic valve function and incident aortic stenosis risk after MTAG in *All of Us*

Standard deviation scaled PRS as predictor

PRS	Hazard ratio for incident AS	P Value
Aortic Stenosis MTAG	1.64 (1.50–1.78)	8.7E-30
AVA MTAG	0.71 (0.65–0.77)	1.1E-15
Peak velocity MTAG	1.53 (1.41–1.67)	3.7E-23
Mean gradient MTAG	1.53 (1.40–1.66)	1.1E-22

PRS status as predictor (Top 5% vs others)

PRS	Hazard ratio for incident AS	P Value
Aortic Stenosis MTAG	3.32 (2.60–4.24)	8.8E-22
AVA MTAG Bottom 5% vs others	0.79 (0.52–1.21)	2.8E-01
Peak velocity MTAG	2.51 (1.92–3.28)	2.1E-11
Mean gradient MTAG	2.61 (2.00–3.40)	1.2E-12

Polygenic predictions of aortic stenosis, aortic valve area (AVA), peak velocity, and mean gradient from summary statistics after MTAG were constructed using 1.1 million HapMap3 SNPs with PRS-cs and residualized using the first 20 principal components of genetic ancestry. They were tested in *All of Us* using Cox proportional hazard models. After exclusion of prevalent aortic stenosis (AS) cases at the time of DNA sampling, 496 cases of incident AS and 243,954 controls contributed to survival analyses with the follow-up time scale. Covariates included self-reported sex, age at DNA collection, age^2 at DNA collection, and the first five principal components of genetic ancestry. Results are shown separately for standard deviation scaled and percentile-stratified PRS as predictors.

Supplementary Table 14: Polygenic predictions of aortic valve function and incident aortic stenosis risk after MTAG in Mass General Brigham Biobank

Standard deviation scaled PRS as predictor

PRS	Hazard ratio for incident AS	P Value
Aortic Stenosis MTAG	1.61 (1.50-1.73)	6.3E-36
AVA MTAG	0.72 (0.67–0.78)	3.5E-17
Peak velocity MTAG	1.56 (1.45–1.69)	6.1E-32
Mean gradient MTAG	1.56 (1.44–1.68)	3.7E-31

PRS status as predictor (Top 5% vs others)

PRS	Hazard ratio for incident AS	P Value
Aortic Stenosis MTAG	2.76 (2.14–3.57)	7.8E-15
AVA MTAG Bottom 5% vs others	2.10 (1.59–2.76)	1.3E-07
Peak velocity MTAG	3.02 (2.36–3.85)	6.3E-19
Mean gradient MTAG	2.79 (2.16–3.60)	4.3E-15

Polygenic predictions of aortic stenosis, aortic valve area (AVA), peak velocity, and mean gradient from summary statistics after MTAG were constructed using 1.1 million HapMap3 SNPs with PRSCs and tested in Mass General Brigham Biobank using Cox proportional hazard models. After exclusion of prevalent aortic stenosis (AS) cases at the time of DNA sampling, 680 cases of incident AS and 42,328 controls contributed to survival analyses with the follow-up time scale. Covariates included self-reported sex, age at DNA collection, age^2 at DNA collection, and the first five principal components of genetic ancestry. Principal components 1 to 10 were regressed out of the PRS. Results are shown separately for standard deviation scaled and percentile-stratified PRS as predictors.

Supplementary Table 15: MAGMA gene set analysis (Excel)

Supplementary Table 16: Overview of performance of lipid based polygenic scores to predict lipid levels (Excel)

Supplementary Table 17: Performance of the lipid polygenic scores to predict continuous aortic valve traits (Excel)

Supplementary Table 18: Mendelian Randomization of risk factors and continuous aortic valve traits (Excel)

Supplementary Table 19: Mendelian Randomization of risk factors and aortic stenosis in METAL meta-analysis before MTAG (Excel)

Supplementary Table 20: Aortic stenosis definition in Mass General Biobank (Excel)

Supplementary Table 21: FinnGen consortium members (Excel)

Supplementary Figures

Supplementary Figure 1: Flow diagram

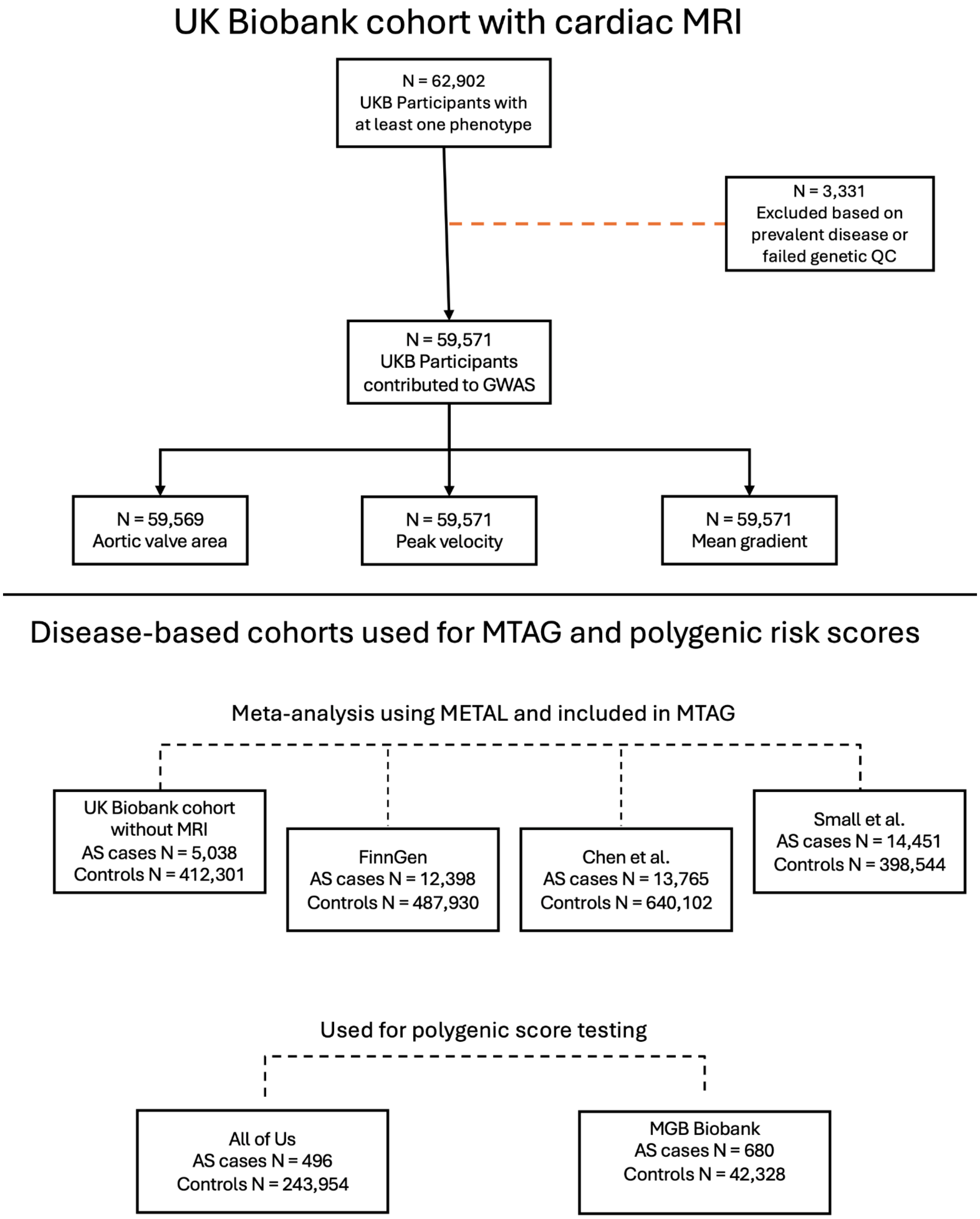

Consort flow chart of cohort construction showing the number of participants with available image series, the overall number of quality control failures and the number of participants that contributed to each respective phenotype genome wide-association study.

Supplementary Figure 2: Manhattan plot of GWAS results for aortic valve traits before MTAG

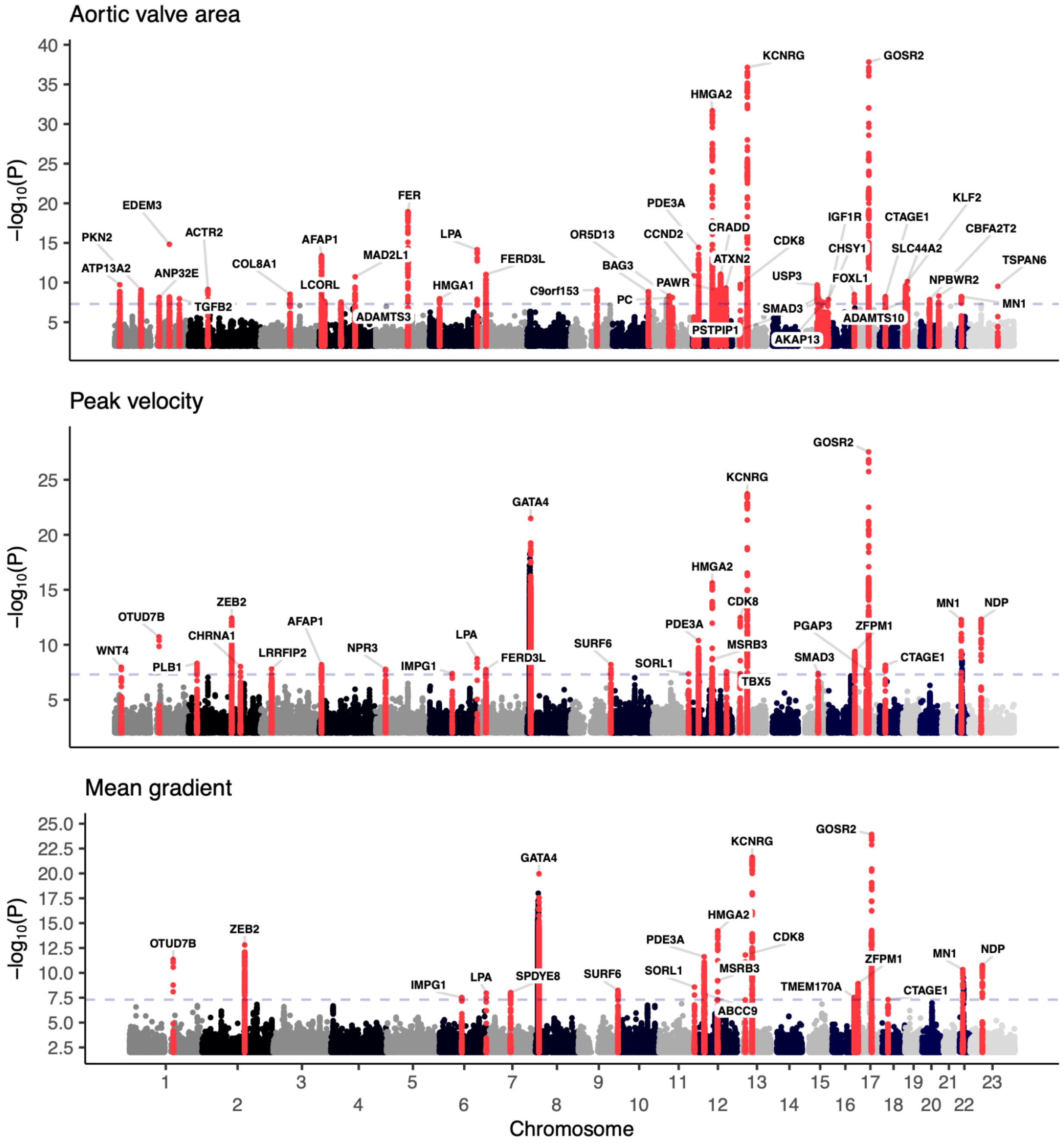

Manhattan plots show the chromosomal position (x axis) and the strength of association (–log10 of the P value, y axis) for MRi-derived traits. The Xchromosome is not represented. Loci that contain SNPs with $P<5\times10^{-8}$ were labeled with the name of the nearest gene; genes may be represented multiple times for the same trait when multiple variants at the same locus are in

linkage equilibrium with one another ($r2 < 0.001$). The analysis was adjusted for sex, age and age^2 at the time of MRI, the genotyping array, the MRI scanner's unique identifier and the first ten principal components of genetic ancestry that were centrally computed by UK Biobank. Loci were colored red if they were associated with the respective trait at $P < 5 \times 10{-}8$. SNPs with $P > 0.01$ are not plotted.

Supplementary Figure 3: Variant effect alignment at MTAG loci

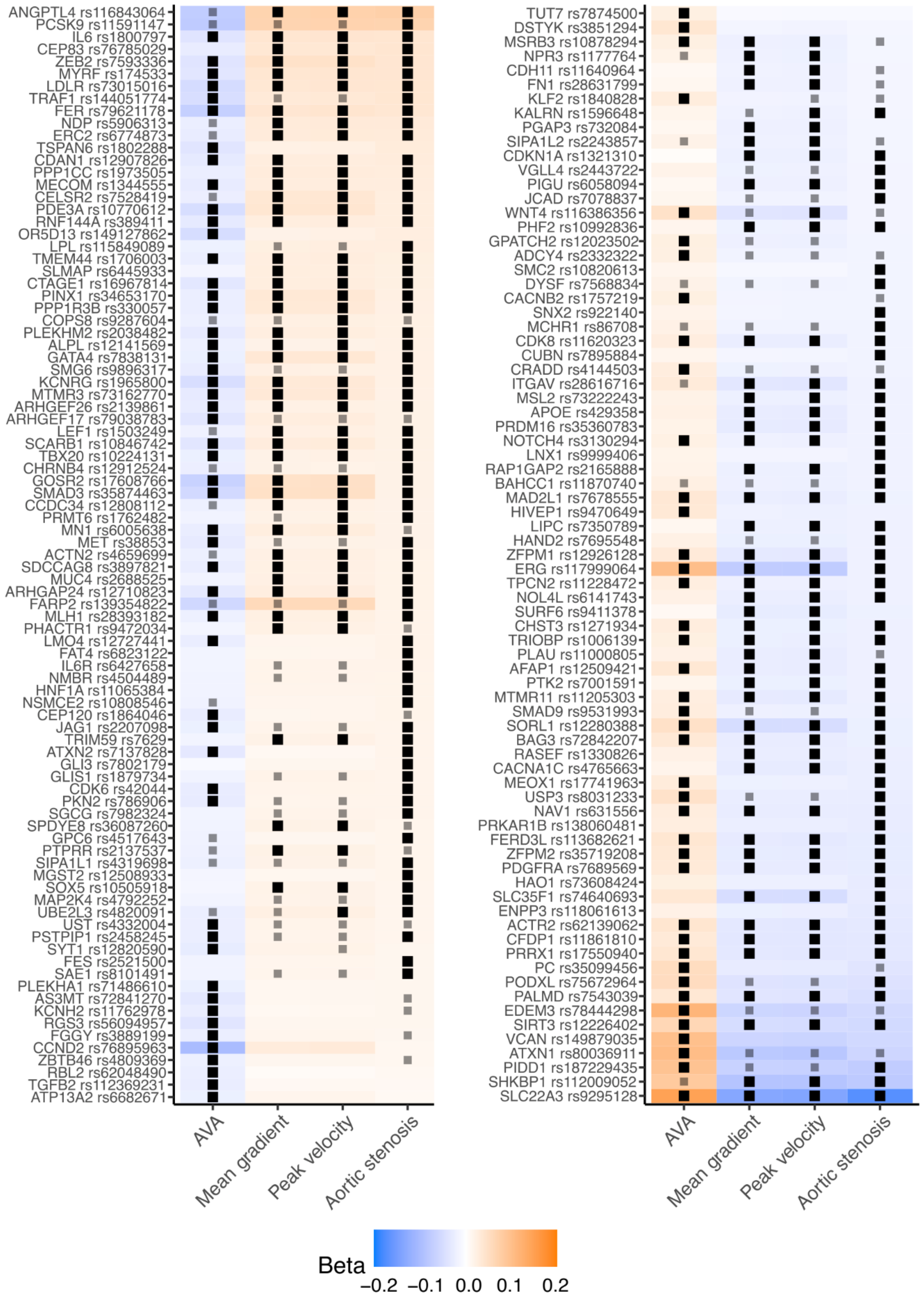

Depiction of the loci significant for at least one trait in MTAG analysis. Effect estimates and P-values are taken from the MTAG analysis. Effect direction is depicted with respect to the reference allele. Variants with a risk-decreasing reference allele for aortic stenosis are depicted in the left panel; those with a risk-increasing reference allele are depicted in the right panel. Within each panel, variants are sorted by effect size for aortic stenosis. Effect sizes have units of standard deviation for AVA, peak velocity, mean gradient, and log odds for aortic stenosis. Variants are represented by nearest gene name and variant identifier; multiple variants at one locus are listed when the lead variant differs across phenotypes. Light gray boxes indicate 5E-06 > P >= 5E-08; dark gray boxes indicate P < 5E-08.

Supplementary Figure 4: Manhattan plot of MAGMA results for aortic valve traits

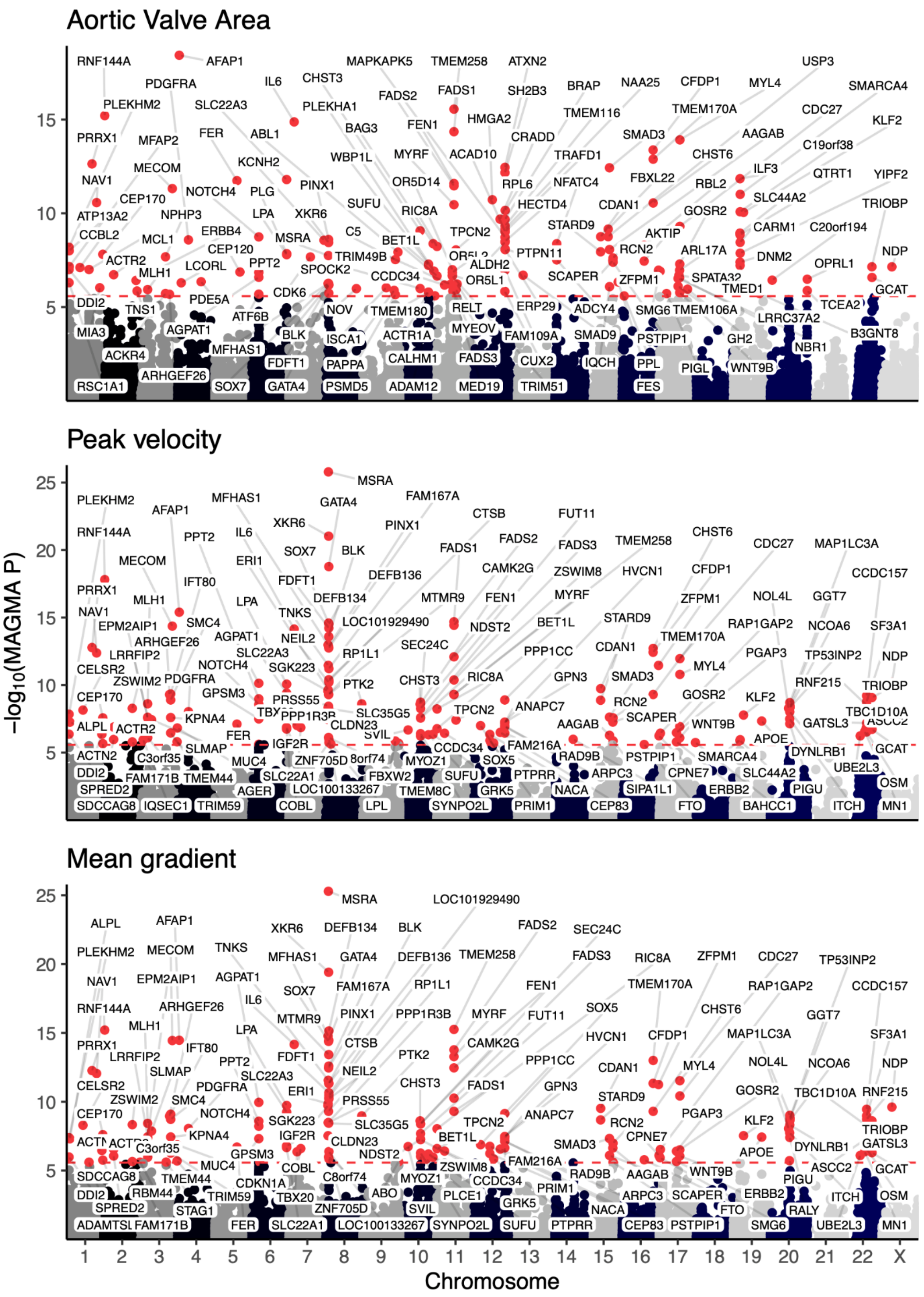

Statistical gene set prioritization for each aortic valve trait by applying the MAGMA framework to the MTAG-augmented GWAS summary statistics. Genes and gene sets were considered significant based on a per-trait false discovery rate < 0.05 using *p.adjust* in R. All tests were two-tailed.

Supplementary Figure 5: Manhattan plot of MAGMA results for aortic stenosis

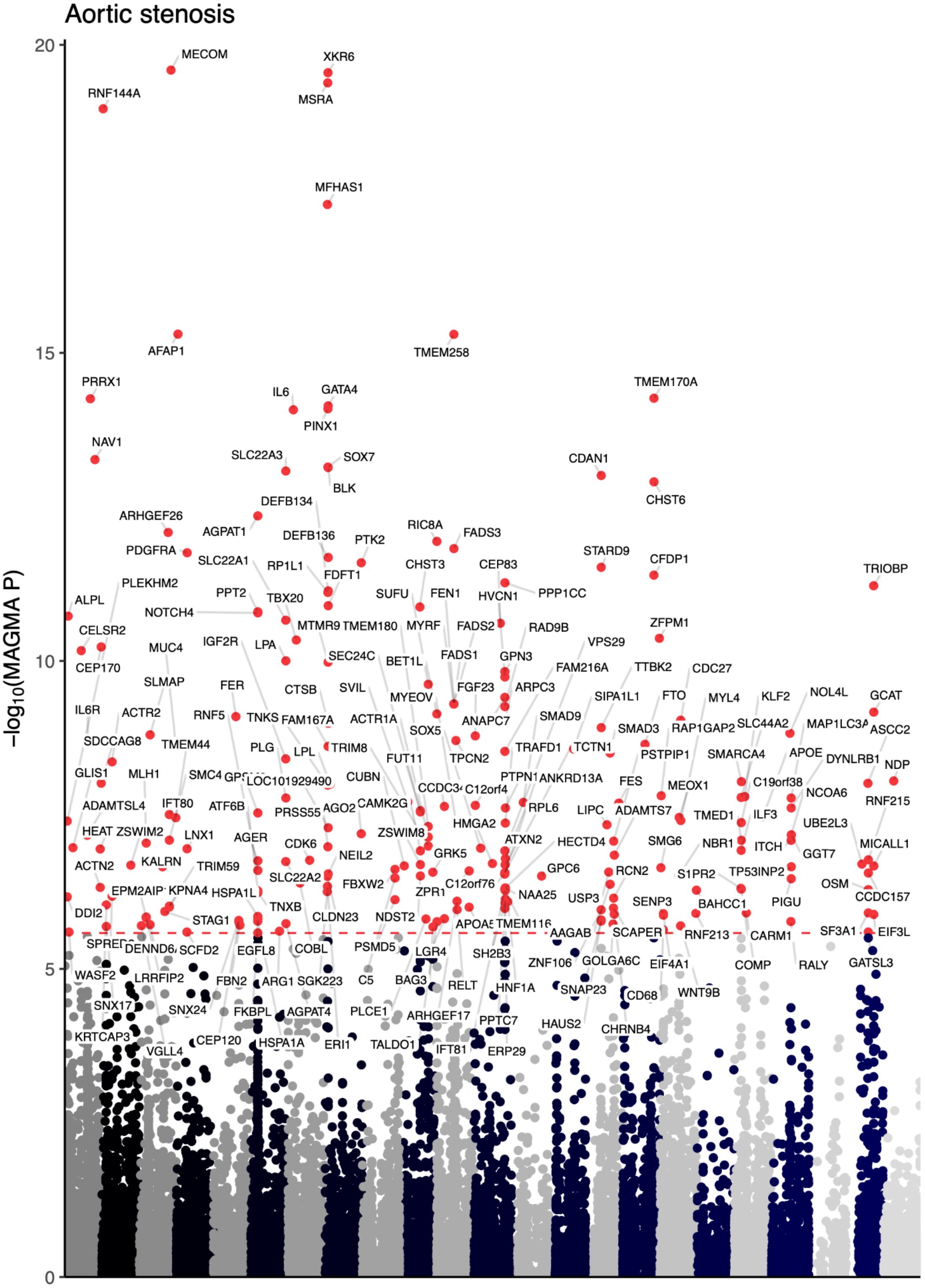

Statistical gene set prioritization for each aortic stenosis GWAS by applying the MAGMA framework to the MTAG-augmented GWAS summary statistics. Genes and gene sets were considered significant based on a per-trait false discovery rate < 0.05 using *p.adjust* in R. All tests were two-tailed.

Supplementary Figure 6: GTEx tissue enrichment

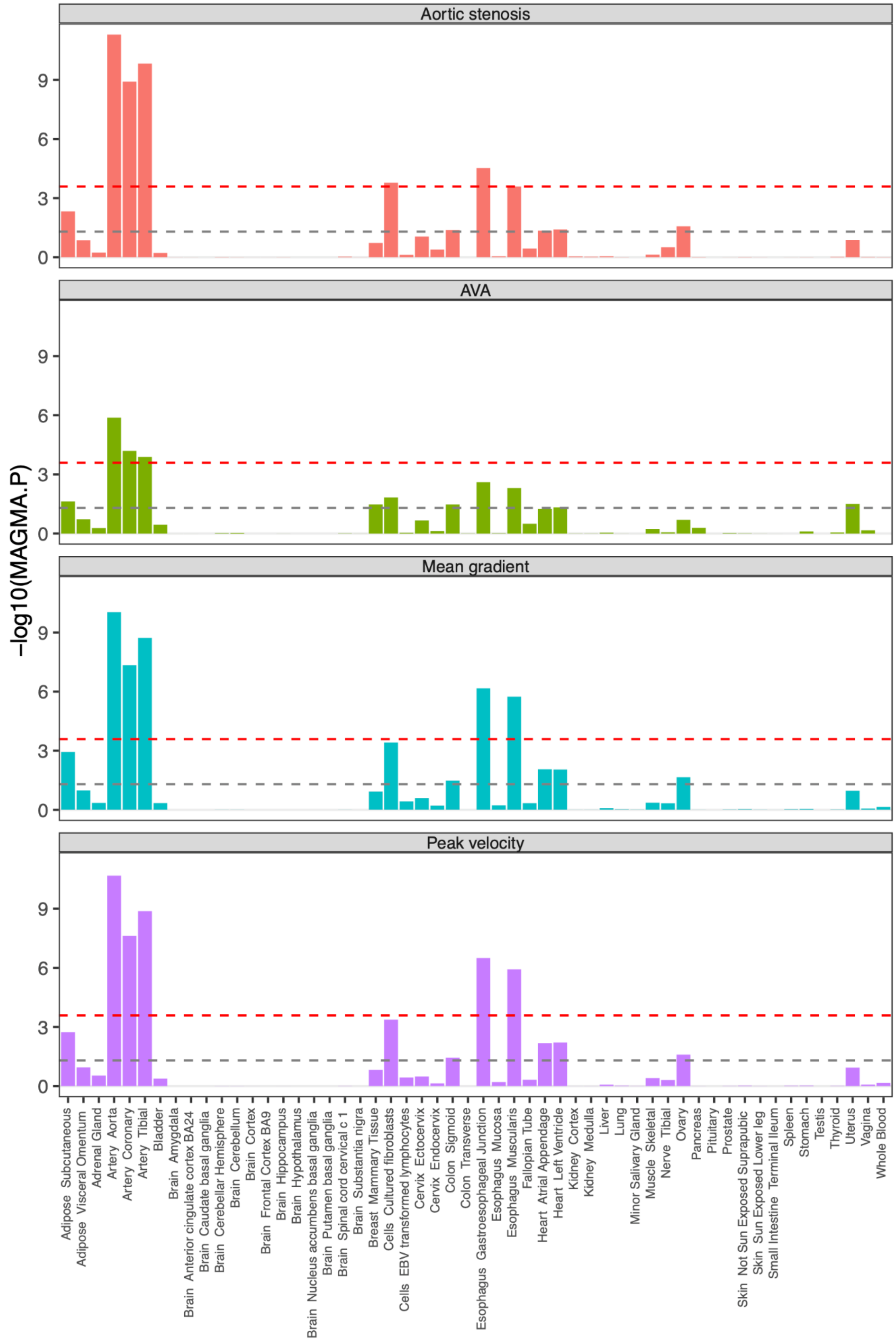

Enrichment of genes from MAGMA analysis of the four GWAS with expression in tissues from GTEx v8. The GTEx tissues are on the x-axis and the -log10(P) value for enrichment of the respective GWAS with the GTEx tissue is on the y-axis. The dashed gray line indicates P = 0.05, while the dashed red line indicates the P value threshold for study-wise significance based on a false discovery rate of 0.05. All tests were two-tailed.

Supplementary Figure 7: aortic valve cell type enrichment

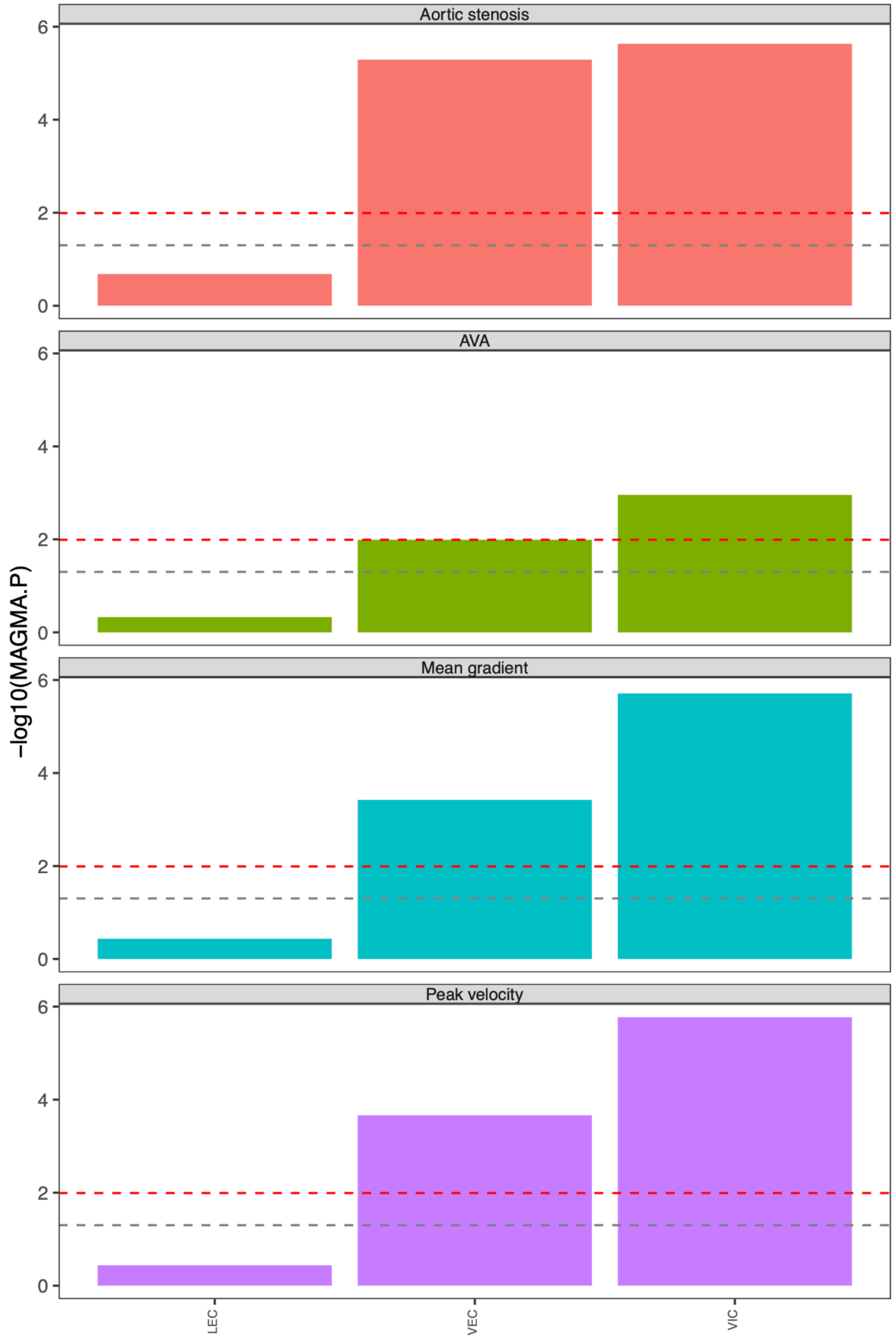

Enrichment of genes from MAGMA analysis of the five GWAS with expression in murine aortic valve cell types from Lee et al[65]. The cell types are on the x-axis and the -log10(P) value for

enrichment of the respective GWAS with the cell type is on the y-axis. The dashed gray line indicates P = 0.05, while the dashed red line indicates the P value threshold for study-wise significance based on a false discovery rate of 0.05. All tests were two-tailed. LEC: Leukocytes; VEC: Valvular Endothelial Cells; VIC: Valvular Interstitial Cells

Supplementary Figure 8: Thoracic aorta cell type enrichment

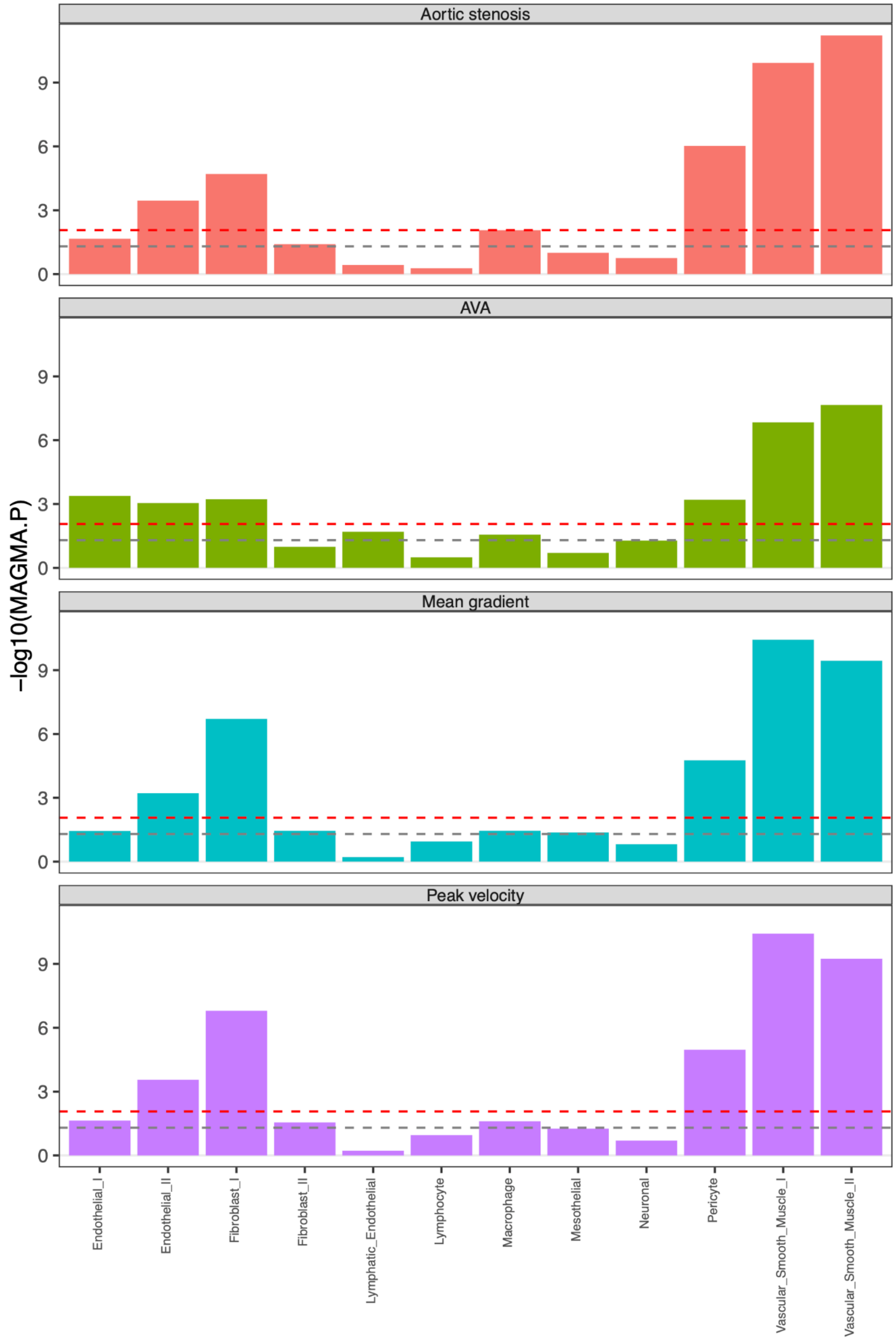

Enrichment of genes from MAGMA analysis of the four GWAS with expression in thoracic aorta cell types from Pirrucello et al[37]. The cell types are on the x-axis and the -log10(P) value for

enrichment of the respective GWAS with the cell type is on the y-axis. The dashed gray line indicates P = 0.05, while the dashed red line indicates the P value threshold for study-wise significance based on a false discovery rate of 0.05. All tests were two-tailed.

Supplementary Figure 9: Left ventricular cell type enrichment

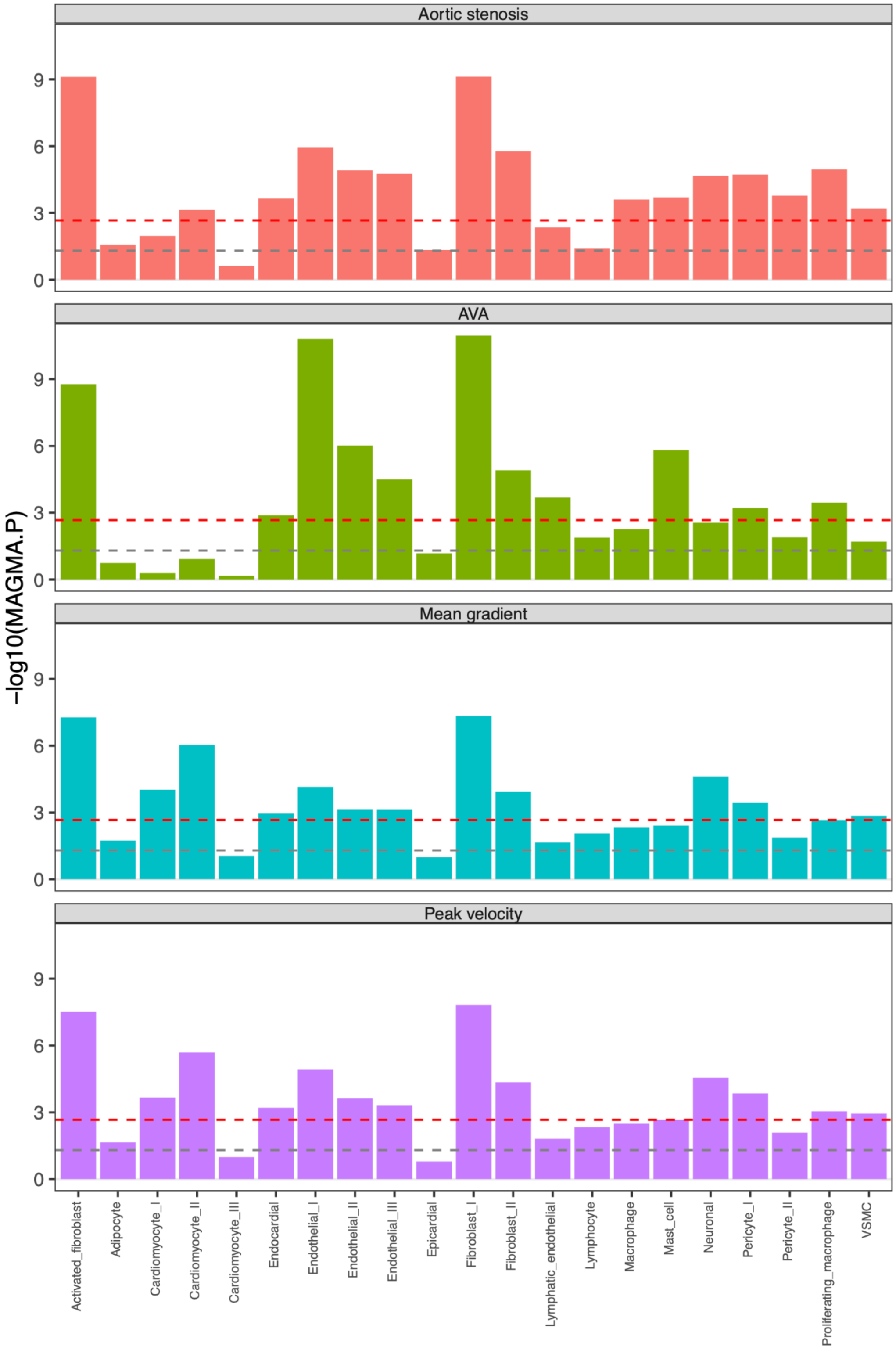

Enrichment of genes from MAGMA analysis of the four GWAS with expression in left ventricular cell types from Chaffin et al[48]. The cell types are on the x-axis and the -log10(P) value for

enrichment of the respective GWAS with the cell type is on the y-axis. The dashed gray line indicates P = 0.05, while the dashed red line indicates the P value threshold for study-wise significance based on a false discovery rate of 0.05. All tests were two-tailed.

References

1. Loh, P.-R. *et al.* Efficient Bayesian mixed model analysis increases association power in large cohorts. Preprint at https://doi.org/10.1101/007799.
2. Vahanian, A. *et al.* 2021 ESC/EACTS Guidelines for the management of valvular heart disease: developed by the Task Force for the management of valvular heart disease of the European Society of Cardiology (ESC) and the European Association for Cardio-Thoracic Surgery (EACTS). *Eur. Heart J.* **43**, 561–632 (2022).
3. Otto, C. M. *et al.* 2020 ACC/AHA Guideline for the Management of Patients With Valvular Heart Disease: A Report of the American College of Cardiology/American Heart Association Joint Committee on Clinical Practice Guidelines. *J. Am. Coll. Cardiol.* **77**, e25–e197 (2021).
4. Helgadottir, A. *et al.* Genome-wide analysis yields new loci associating with aortic valve stenosis. *Nat. Commun.* **9**, 987 (2018).
5. Yang, B. *et al.* Protein-altering and regulatory genetic variants near GATA4 implicated in bicuspid aortic valve. *Nat. Commun.* **8**, 15481 (2017).
6. Siu, S. C. & Silversides, C. K. Bicuspid aortic valve disease. *J. Am. Coll. Cardiol.* **55**, 2789–2800 (2010).
7. Fazel, S. S. *et al.* The aortopathy of bicuspid aortic valve disease has distinctive patterns and usually involves the transverse aortic arch. *J. Thorac. Cardiovasc. Surg.* **135**, 901–7, 907.e1–2 (2008).
8. Córdova-Palomera, A. *et al.* Cardiac imaging of aortic valve area from 34 287 UK Biobank participants reveals novel genetic associations and shared genetic comorbidity with multiple

disease phenotypes. *Circ. Genom. Precis. Med.* **13**, e003014 (2020).

9. Lynch, S. A. *et al.* The 12q14 microdeletion syndrome: six new cases confirming the role of HMGA2 in growth. *Eur. J. Hum. Genet.* **19**, 534–539 (2011).
10. van de Laar, I. M. B. H. *et al.* Mutations in SMAD3 cause a syndromic form of aortic aneurysms and dissections with early-onset osteoarthritis. *Nat. Genet.* **43**, 121–126 (2011).
11. MacFarlane, E. G. *et al.* Lineage-specific events underlie aortic root aneurysm pathogenesis in Loeys-Dietz syndrome. *J. Clin. Invest.* **129**, 659–675 (2019).
12. Aragam, K. G. *et al.* Discovery and systematic characterization of risk variants and genes for coronary artery disease in over a million participants. *Nat. Genet.* **54**, 1803–1815 (2022).
13. Ma, L. *et al.* The HDAC9-associated risk locus promotes coronary artery disease by governing TWIST1. *PLoS Genet.* **18**, e1010261 (2022).
14. Takahashi, M. *et al.* Enhanced circulating soluble LR11 in patients with coronary organic stenosis. *Atherosclerosis* **210**, 581–584 (2010).
15. Hinton, R. B. *et al.* Elastin haploinsufficiency results in progressive aortic valve malformation and latent valve disease in a mouse model. *Circ. Res.* **107**, 549–557 (2010).
16. Wei, W.-Q. *et al.* LPA Variants Are Associated With Residual Cardiovascular Risk in Patients Receiving Statins. *Circulation* **138**, 1839–1849 (2018).
17. Lee, J. S. *et al.* Klf2 is an essential regulator of vascular hemodynamic forces in vivo. *Dev. Cell* **11**, 845–857 (2006).
18. Rasouli, S. J. *et al.* The flow responsive transcription factor Klf2 is required for myocardial wall integrity by modulating Fgf signaling. *Elife* **7**, (2018).
19. Jin, Y.-J. *et al.* Protein kinase N2 mediates flow-induced endothelial NOS activation and vascular tone regulation. *J. Clin. Invest.* **131**, (2021).
20. Gan, L. *et al.* Ischemic Heart-Derived Small Extracellular Vesicles Impair Adipocyte Function. *Circ. Res.* **130**, 48–66 (2022).

21. Erdmann, J., Kessler, T., Munoz Venegas, L. & Schunkert, H. A decade of genome-wide association studies for coronary artery disease: the challenges ahead. *Cardiovasc. Res.* **114**, 1241–1257 (2018).

22. Hartiala, J. A. *et al.* Genome-wide analysis identifies novel susceptibility loci for myocardial infarction. *Eur. Heart J.* **42**, 919–933 (2021).

23. Lindsay, M. E. *et al.* Loss-of-function mutations in TGFB2 cause a syndromic presentation of thoracic aortic aneurysm. *Nat. Genet.* **44**, 922–927 (2012).

24. Pirruccello, J. P. *et al.* The Genetic Determinants of Aortic Distention. *J. Am. Coll. Cardiol.* **81**, 1320–1335 (2023).

25. Skrbic, B. *et al.* Lack of collagen VIII reduces fibrosis and promotes early mortality and cardiac dilatation in pressure overload in mice. *Cardiovasc. Res.* **106**, 32–42 (2015).

26. Zhu, W., Zhao, M., Mattapally, S., Chen, S. & Zhang, J. CCND2 Overexpression Enhances the Regenerative Potency of Human Induced Pluripotent Stem Cell–Derived Cardiomyocytes. *Circ. Res.* **122**, 88–96 (2018).

27. Zakhary, D. R., Moravec, C. S. & Bond, M. Regulation of PKA binding to AKAPs in the heart: alterations in human heart failure. *Circulation* **101**, 1459–1464 (2000).

28. Algül, S. *et al.* EGFR/IGF1R Signaling Modulates Relaxation in Hypertrophic Cardiomyopathy. *Circ. Res.* **133**, 387–399 (2023).

29. Franaszczyk, M. *et al.* The BAG3 gene variants in Polish patients with dilated cardiomyopathy: four novel mutations and a genotype-phenotype correlation. *J. Transl. Med.* **12**, 192 (2014).

30. Ge, T., Chen, C.-Y., Ni, Y., Feng, Y.-C. A. & Smoller, J. W. Polygenic prediction via Bayesian regression and continuous shrinkage priors. *Nat. Commun.* **10**, 1776 (2019).

31. International HapMap 3 Consortium *et al.* Integrating common and rare genetic variation in diverse human populations. *Nature* **467**, 52–58 (2010).

32. Baumgartner, H., Chair *et al.* Recommendations on the echocardiographic assessment of

aortic valve stenosis: a focused update from the European Association of Cardiovascular Imaging and the American Society of Echocardiography. *Eur. Heart J. Cardiovasc. Imaging* **18**, 254–275 (2017).

33. Sudlow, C. *et al.* UK biobank: an open access resource for identifying the causes of a wide range of complex diseases of middle and old age. *PLoS Med.* **12**, e1001779 (2015).
34. Bycroft, C. *et al.* The UK Biobank resource with deep phenotyping and genomic data. *Nature* **562**, 203–209 (2018).
35. Raisi-Estabragh, Z., Harvey, N. C., Neubauer, S. & Petersen, S. E. Cardiovascular magnetic resonance imaging in the UK Biobank: a major international health research resource. *Eur. Heart J. Cardiovasc. Imaging* **22**, 251–258 (2021).
36. Petersen, S. E. *et al.* UK Biobank's cardiovascular magnetic resonance protocol. *J. Cardiovasc. Magn. Reson.* **18**, (2015).
37. Pirruccello, J. P. *et al.* Deep learning enables genetic analysis of the human thoracic aorta. *Nat. Genet.* **54**, 40–51 (2022).
38. Nyul, L. G., Udupa, J. K. & Zhang, X. New variants of a method of MRI scale standardization. *IEEE Transactions on Medical Imaging* vol. 19 143–150 Preprint at https://doi.org/10.1109/42.836373 (2000).
39. Shinohara, R. T. *et al.* Statistical normalization techniques for magnetic resonance imaging. *Neuroimage Clin* **6**, 9–19 (2014).
40. Riba, E., Mishkin, D., Ponsa, D., Rublee, E. & Bradski, G. Kornia: an Open Source Differentiable Computer Vision Library for PyTorch. *arXiv [cs.CV]* 3674–3683 (2019).
41. Smith, L. N. & Topin, N. Super-convergence: very fast training of neural networks using large learning rates. in *Artificial Intelligence and Machine Learning for Multi-Domain Operations Applications* vol. 11006 369–386 (SPIE, 2019).
42. Lin, T.-Y., Goyal, P., Girshick, R., He, K. & Dollar, P. Focal Loss for Dense Object Detection. *IEEE Trans. Pattern Anal. Mach. Intell.* **42**, 318–327 (2020).

43. Kirillov, A., Mintun, E., Ravi, N., Mao, H. & Rolland, C. Segment anything. *arXiv preprint arXiv* (2023).

44. Horn, B., Klaus, B. & Horn, P. *Robot Vision*. (MIT Press, 1986).

45. Yap, S.-C. *et al.* A simplified continuity equation approach to the quantification of stenotic bicuspid aortic valves using velocity-encoded cardiovascular magnetic resonance. *J. Cardiovasc. Magn. Reson.* **9**, 899–906 (2007).

46. de Leeuw, C. A., Mooij, J. M., Heskes, T. & Posthuma, D. MAGMA: generalized gene-set analysis of GWAS data. *PLoS Comput. Biol.* **11**, e1004219 (2015).

47. Lonsdale, J. *et al.* The Genotype-Tissue Expression (GTEx) project. *Nat. Genet.* **45**, 580–585 (2013).

48. Chaffin, M. *et al.* Single-nucleus profiling of human dilated and hypertrophic cardiomyopathy. *Nature* **608**, 174–180 (2022).

49. Ma, W. F. *et al.* PlaqView 2.0: A comprehensive web portal for cardiovascular single-cell genomics. *Front Cardiovasc Med* **9**, 969421 (2022).

50. Robinson, M. D., McCarthy, D. J. & Smyth, G. K. edgeR: a Bioconductor package for differential expression analysis of digital gene expression data. *Bioinformatics* **26**, 139–140 (2010).

51. Law, C. W., Chen, Y., Shi, W. & Smyth, G. K. voom: Precision weights unlock linear model analysis tools for RNA-seq read counts. *Genome Biol.* **15**, R29 (2014).

52. Investigators, T. A. of U. R. P. & The All of Us Research Program Investigators. The 'All of Us' Research Program. *New England Journal of Medicine* vol. 381 668–676 Preprint at https://doi.org/10.1056/nejmsr1809937 (2019).

53. Venner, E. *et al.* Whole-genome sequencing as an investigational device for return of hereditary disease risk and pharmacogenomic results as part of the All of Us Research Program. *Genome Med.* **14**, 34 (2022).

54. Hinrichs, A. S. *et al.* The UCSC Genome Browser Database: update 2006. *Nucleic Acids*

Res. **34**, D590–8 (2006).

55. Chang, C. C. *et al.* Second-generation PLINK: rising to the challenge of larger and richer datasets. Gigascience. 2015; 4: 7. Preprint at (2015).
56. Kurki, M. I. *et al.* FinnGen provides genetic insights from a well-phenotyped isolated population. *Nature* **613**, 508–518 (2023).
57. Karlson, E. W., Boutin, N. T., Hoffnagle, A. G. & Allen, N. L. Building the Partners HealthCare Biobank at partners personalized medicine: Informed consent, return of research results, recruitment lessons and operational considerations. *J. Pers. Med.* **6**, 2 (2016).
58. Boutin, N. T. *et al.* Implementation of Electronic Consent at a Biobank: An Opportunity for Precision Medicine Research. *J Pers Med* **6**, (2016).
59. Jurgens, S. J. *et al.* Analysis of rare genetic variation underlying cardiometabolic diseases and traits among 200,000 individuals in the UK Biobank. *Nat. Genet.* **54**, 240–250 (2022).
60. Conomos, M. P., Reiner, A. P., Weir, B. S. & Thornton, T. A. Model-free Estimation of Recent Genetic Relatedness. *Am. J. Hum. Genet.* **98**, 127–148 (2016).
61. Conomos, M. P., Miller, M. B. & Thornton, T. A. Robust inference of population structure for ancestry prediction and correction of stratification in the presence of relatedness. *Genet. Epidemiol.* **39**, 276–293 (2015).
62. 1000 Genomes Project Consortium *et al.* A global reference for human genetic variation. *Nature* **526**, 68–74 (2015).
63. Taliun, D. *et al.* Sequencing of 53,831 diverse genomes from the NHLBI TOPMed Program. *Nature* **590**, 290–299 (2021).
64. Gaziano, J. M. *et al.* Million Veteran Program: A mega-biobank to study genetic influences on health and disease. *J. Clin. Epidemiol.* **70**, 214–223 (2016).
65. Lee, S. H. *et al.* Single-cell transcriptomics reveal cellular diversity of aortic valve and the immunomodulation by PPARγ during hyperlipidemia. *Nat. Commun.* **13**, 5461 (2022).
