# Supplementary material for: Multitrait analyses identify genetic variants associated with aortic valve function and aortic stenosis risk
Source: Nat Genet. 2025 Dec 19;58(1):47–56. doi: 10.1038/s41588-025-02397-7 (PMC12807864; doi:10.1038/s41588-025-02397-7)
Supplement: Supplementary file 2 — Reporting Summary [file 41588_2025_2397_MOESM2_ESM.pdf]

nature portfolio

Corresponding author(s): James P. Pirruccello
Last updated by author(s): Feb 24, 2025

Reporting Summary

Nature Portfolio wishes to improve the reproducibility of the work that we publish. This form provides structure for consistency and transparency in reporting. For further information on Nature Portfolio policies, see our Editorial Policies and the Editorial Policy Checklist.

Statistics

For all statistical analyses, confirm that the following items are present in the figure legend, table legend, main text, or Methods section.

n/a	Confirmed	
☐	☒	The exact sample size (*n*) for each experimental group/condition, given as a discrete number and unit of measurement
☐	☒	A statement on whether measurements were taken from distinct samples or whether the same sample was measured repeatedly
☐	☒	The statistical test(s) used AND whether they are one- or two-sided<br>*Only common tests should be described solely by name; describe more complex techniques in the Methods section.*
☐	☒	A description of all covariates tested
☐	☒	A description of any assumptions or corrections, such as tests of normality and adjustment for multiple comparisons
☐	☒	A full description of the statistical parameters including central tendency (e.g. means) or other basic estimates (e.g. regression coefficient) AND variation (e.g. standard deviation) or associated estimates of uncertainty (e.g. confidence intervals)
☐	☒	For null hypothesis testing, the test statistic (e.g. *F*, *t*, *r*) with confidence intervals, effect sizes, degrees of freedom and *P* value noted<br>*Give P values as exact values whenever suitable.*
☒	☐	For Bayesian analysis, information on the choice of priors and Markov chain Monte Carlo settings
☒	☐	For hierarchical and complex designs, identification of the appropriate level for tests and full reporting of outcomes
☐	☒	Estimates of effect sizes (e.g. Cohen's *d*, Pearson's *r*), indicating how they were calculated

Our web collection on statistics for biologists contains articles on many of the points above.

Software and code

Policy information about availability of computer code

Data collection	PyTorch v.2.1.0<br>TraceOverlay v0.1.0
Data analysis	R4.2.2<br>BOLT-REML v2.3.4<br>REGENIE v2.2.4<br>METAL v2020-05-05<br>MAGMA version 1.09b<br>MTAG 1.0.8<br>GSEA v4.3.2<br>PRScs v1.1.0<br>Two SampleMR v.0.4.9

For manuscripts utilizing custom algorithms or software that are central to the research but not yet described in published literature, software must be made available to editors and reviewers. We strongly encourage code deposition in a community repository (e.g. GitHub). See the Nature Portfolio guidelines for submitting code & software for further information.

Data

Policy information about availability of data

All manuscripts must include a data availability statement. This statement should provide the following information, where applicable:

- Accession codes, unique identifiers, or web links for publicly available datasets
- A description of any restrictions on data availability
- For clinical datasets or third party data, please ensure that the statement adheres to our policy

Aortic valve measurements have been returned to the UK Biobank for access by researchers with UK Biobank access. Summary statistics for the GWAS meta-analyses have been deposited into the GWAS Catalog as well as to Zenodo (DOI 10.5281/zenodo.14025285(Kany and Pirruccello 2024)). The polygenic score weights are available on Zenodo (DOI 10.5281/zenodo.15069071(Kany and Pirruccello 2025)) and in the PGS Catalog. Individual-level data from Finnish biobanks can be accessed through the Fingenious® services (https://site.fingenious.fi/en/) managed by FINBB. Finnish Health register data can be applied from Findata (https://findata.fi/en/data/). The All of Us biobank access is currently granted to researchers at academic, non-profit, and certain for-profit health institutions, with plans to expand. Researchers register through the All of Us Researcher Workbench (including identity verification and training) and must comply with Data Use Policies. Research projects using these data are publicly listed in the All of Us Research Projects Directory. Data from the MGB Biobank are available to researchers affiliated with MGB who have approval. It is currently not possible to access the MGB Biobank without a MGB affiliation. Further information regarding access can be obtained through email to biobank@partners.org. MVP aortic stenosis summary statistics are available from dbGaP under accession #phs001672. The Chen 2023 aortic stenosis summary statistics are available at https://zenodo.org/records/7829401 .

Research involving human participants, their data, or biological material

Policy information about studies with human participants or human data. See also policy information about sex, gender (identity/presentation), and sexual orientation and race, ethnicity and racism.

Reporting on sex and gender	Sex was determined by genetic and self-reported sex and partcipants retained for analysis if both matched.
Reporting on race, ethnicity, or other socially relevant groupings	No special consideration on race and ethnicity in this work. The first 5 principal components of genetic ancestry were used for adjusting regression analyses.
Population characteristics	Informed consent was obtained from all participants. Comprehensive phenotyping including questionnaires about family history, physical traits, life-style factors, laboratory values and imaging was obtained for each participant. Inpatient electronic health records from Hospital Episode Statistics (England), Patient Episode Database (Wales) and Scottish Morbidity Records (Scotland) as well as National Health Service death registries are linked to the cohort.The imaging substudy of the UK Biobank is planned to perform 1.5 Tesla cardiac MRI in ca. 100,000 participants with ca. 65,000 studies in individual participants available as of the time of manuscript preparation.For the UK Biobank, participants were genotyped with either the UK Biobank Axiom or UK Bi LEVE arrays and imputation conducted with the UKl0K+ 1000 Genomes phase Ill and Haplotype Reference Consortium panel.<br>Out of the 59,571 participants in the UK Biobank, 52% were female, the mean age at the time of MRI was 65.9 years and mean body mass index 26.5 kg/m^2. Mean systolic blood pressure was 141mmHg, and a total of 341 had moderate aortic stenosis and 46 had severe aortic stenosis at baseline.
Recruitment	The UK Biobank is a prospective, general population-based cohort study that enrolled ~500,000 individuals in the UK between the ages 40-69 years from 2006-2010.
Ethics oversight	This project was conducted under UK Biobank application #41664, in the All of Us cohort as well as FinnGen. It was considered exempt by the UCSF IRB (#22-37715), and approved by the MGB institutional review board (IRB; protocol 2019P003144).

Note that full information on the approval of the study protocol must also be provided in the manuscript.

Field-specific reporting

Please select the one below that is the best fit for your research. If you are not sure, read the appropriate sections before making your selection.

☒ Life sciences ☐ Behavioural & social sciences ☐ Ecological, evolutionary & environmental sciences

For a reference copy of the document with all sections, see nature.com/documents/nr-reporting-summary-flat.pdf

Life sciences study design

All studies must disclose on these points even when the disclosure is negative.

Sample size	All available participants with cardiac imaging at the time of the study were included. 62,902 participants with deep -earning based flow measurements. A total of 3,331 participants were excluded for pre-existing cardiovascular disease or genetic quality control, leaving 59,571 participants contributing to the GWAS of peak velocity and mean gradient, and 59,569 contributing to GWAS of AVA.

Data exclusions	Participants were excluded if they failed the genetic quality control or had preexisting cardiovascular disease.
Replication	The GWAS of aortic valve function itself could not be replicated due to the lack of a similar cohort with cardiac imaging and lack of indication bias. The GWAS were used to create polygenic risk scores that were replicated in FinnGen and All of Us.
Randomization	This was a genetic study using continuous traits of a observational cohort study.
Blinding	Since there is no treatment investigated here, blinding does not apply.

Reporting for specific materials, systems and methods

We require information from authors about some types of materials, experimental systems and methods used in many studies. Here, indicate whether each material, system or method listed is relevant to your study. If you are not sure if a list item applies to your research, read the appropriate section before selecting a response.

Materials & experimental systems

n/a	Involved in the study
☒	☐ Antibodies
☒	☐ Eukaryotic cell lines
☒	☐ Palaeontology and archaeology
☒	☐ Animals and other organisms
☒	☐ Clinical data
☒	☐ Dual use research of concern
☒	☐ Plants

Methods

n/a	Involved in the study
☒	☐ ChIP-seq
☒	☐ Flow cytometry
☒	☐ MRI-based neuroimaging

Plants

Seed stocks	N/a, this is not a plant study.
Novel plant genotypes	N/a, this is not a plant study.
Authentication	N/a, this is not a plant study.
